# RGD conjugated cell uptake off to on responsive NIR-AZA fluorophores: applications toward intraoperative fluorescence guided surgery†
†Electronic supplementary information (ESI) available: All experimental protocols, data for table entries and images, spectroscopy and analytical data and spectra. See DOI: 10.1039/c9sc02197c


**DOI:** 10.1039/c9sc02197c

**Published:** 2019-06-21

**Authors:** Dan Wu, Harrison C. Daly, Marco Grossi, Emer Conroy, Bo Li, William M. Gallagher, Robert Elmes, Donal F. O'Shea

**Affiliations:** a Department of Chemistry , RCSI , 123 St. Stephen's Green , Dublin 2 , Ireland . Email: donalfoshea@rcsi.ie; b School of Biomolecular and Biomedical Science , Conway Institute, University College Dublin , Belfield , Dublin 4 , Ireland; c Department of Chemistry , Maynooth University Human Health Institute , Maynooth University , Maynooth , Ireland

## Abstract

The use of NIR-fluorescence imaging to demarcate tumour boundaries for real-time guidance of their surgical resection has a huge untapped potential. However, fluorescence imaging using molecular fluorophores, even with a targeting biomolecule attached, has a major shortcoming of signal interference from non-specific background fluorescence outside the region of interest. This poor selectivity necessitates prolonged time delays to allow clearance of background fluorophore and retention within the tumour prior to image acquisition. In this report, an innovative approach to overcome this issue is described in which cancer targeted *off to on* bio-responsive NIR-fluorophores are utilised to switch-on first within the tumour. Bio-responsive cRGD, iRGD and PEG conjugates have been synthesised using activated ester/amine or maleimide/thiol couplings to link targeting and fluorophore components. Their *off to on* emission responses were measured and compared with an *always-on* non-responsive control with each bio-responsive derivative showing large fluorescence enhancement values. Live cell imaging experiments using metastatic breast cancer cells confirmed *in vitro* bio-responsive capabilities. An *in vivo* assessment of MDA-MB 231 tumour imaging performance for bio-responsive and *always-on* fluorophores was conducted with monitoring of fluorescence distributions over 96 h. As anticipated, the *always-on* fluorophore gave an immediate, non-specific and very strong emission throughout whereas the bio-responsive derivatives initially displayed very low fluorescence. All three bio-responsive derivatives switched on within tumours at time points consistent with their conjugated targeting groups. cRGD and iRGD conjugates both had effective tumour turn-on in the first hour, though the cRGD derivative had superior specificity for tumour over the iRGD conjugate. The pegylated derivative had similar switch-on characteristics but over a much longer period, taking 9 h before a significant emission was observable from the tumour. Evidence for *in vivo* active tumour targeting was obtained for the best performing cRGD bio-responsive NIR-AZA derivative from competitive binding studies. Overall, this cRGD-conjugate has the potential to overcome the inherent drawback of targeted *always-on* fluorophores requiring prolonged clearance times and shows excellent potential for clinical translation for intraoperative use in fluorescence guided tumour resections.

## Abstract 2

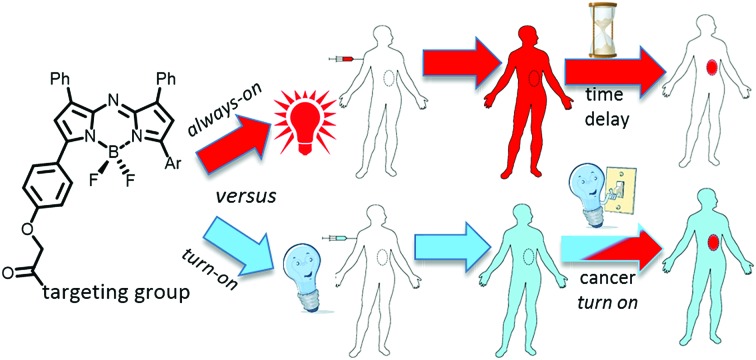
The tumour switches on the NIR-AZA emission for fluorescence guided surgery.

## Introduction

Intraoperative fluorescence imaging to guide surgical resections in real-time has huge untapped potential. Advantages lie in its ease of use, enhanced safety profile over radiolabelling and the ability to acquire image data in real-time during surgical procedures.^1^ Currently indocyanine green (ICG) is the sole clinically approved near infrared red (NIR) fluorophore.^2^ Clinical uses include vascularisation assessments during reconstructive^3^ and bowel anastomoses^4^ surgeries and lymph node mapping in digestive tract,^5^ cervical^6^ and breast^7^ tissues. Due to its non-specificity and very short *in vivo* half-life, its use as an agent to demarcate tumour boundaries for surgical resection is limited to hepatocellular carcinoma of the liver.^8^,^9^

**Fig. 1:**
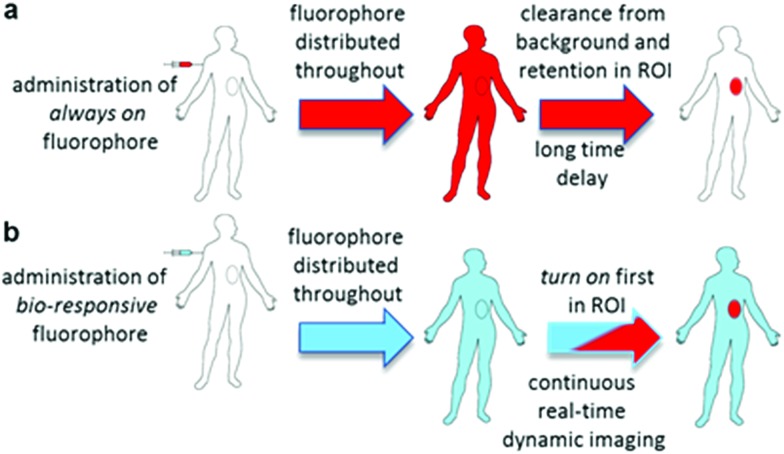
Potential of bio-responsive NIR-fluorescence imaging. (a) Sequence following i.v. administration of *always-on* fluorophore. (b) Sequence following i.v. administration of bio-responsive fluorophore (red indicates fluorescence blue indicated bio-responsive fluorophore turned off).

As a result, new classes of NIR-fluorophore have recently emerged, several of which are bio-conjugated to enhance their affinity for specific cancer types.^10^ However, a remaining complexity for *in vivo* fluorescence imaging using molecular fluorophores exists. Following intravenous administration, fluorophore distributes to all vascularised regions within seconds, resulting in a strong non-specific fluorescence. This necessitates an unpredictable time delay to allow background fluorophore clearance, following which imaging is achievable if sufficient fluorophore is retained in the region of interest (ROI) (Fig. 1a). This limitation is irrespective of whether the fluorophore alone is used or if conjugated to a targeting group (*e.g.* antibody), as an initial broad distribution will still occur. The time between administration and imaging depends on several parameters such as rates of accumulation and clearance from both the ROI and surrounding tissues and elimination from the body *via* metabolic and excretion pathways. Each of these factors can be influenced by the structure of fluorophore itself and by groups conjugated to it, but a time lag before imaging is unavoidable. To provide sufficient contrast for imaging, it is necessary to identify an optimal time point at which a maximum quantity of fluorophore is retained in the ROI with a minimum remaining in the surrounding tissues. For example, antibody conjugated fluorophores have been adopted in recent clinical trials for visualising breast and colorectal cancers utilising labelled bevacizumab and carcinoembryonic antigen (CEA) respectively.^11^,^12^ Yet in spite of using these expensive cancer specific antibody technologies, fluorescence images could only be acquired between two and four days post administration. The prolonged waiting period to achieve sufficient tissue contrast is due to the very long biological half-lives of antibody labelled agents. This time delay adds significant uncertainty to their practical use and raises doubts as to whether all of the cancer would then be detectable by the low levels of remaining fluorophore. In effect, what makes large molecular weight antibodies attractive for sustained drug delivery, can work against them when used for the delivery of contrast agents (Fig. 1a).

Thus, for rapid and accurate intraoperative imaging innovative alternative approaches are needed to enhance target-to-background signal ratio at early stages following fluorophore introduction. One plausible solution is to exploit a mechanism of selective fluorescence quenching in the background areas, whilst first establishing the emitting potential of the fluorophore in the ROI (Fig. 1b). This overcomes the issue of waiting for background clearance and allows observation of dynamic tissue accumulation in real-time during the course of the surgical procedure. While beyond the scope of this work, it may become feasible that dynamic images are recorded continuously, with tissue classifications determined using in-line software image analysis.

**Fig. 2:**
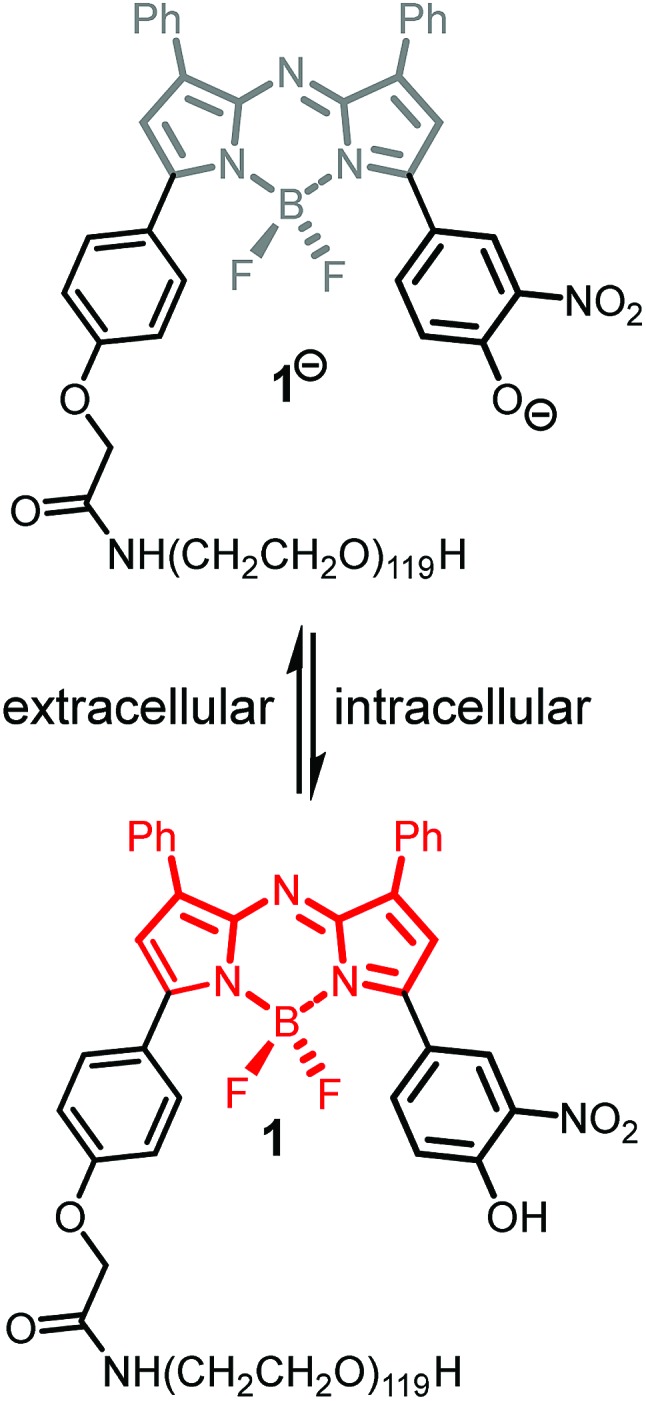
Lysosomal *off*/*on* bio-responsive NIR-AZA fluorophore **1**.

In our previous work, we have shown that bio-responsive NIR-AZA fluorophore **1** performs as an excellent probe capable of real-time continuous imaging of fundamental cellular processes such as endocytosis, lysosomal trafficking and efflux (Fig. 2).13*a* Specifically, the highly photostable NIR fluorescent probe **1** has off/on fluorescence switching controlled by a reversible phenol/phenolate interconversion (Fig. 2). Emission from the probe was shown to be highly selective for cellular lysosomes and, as the off/on switching mechanism is reversible, it is capable of real-time continuous imaging of lysosomal trafficking in 3D or 4D over prolonged time periods without perturbing normal cellular function.^14^ Preliminary *in vivo* imaging in a mouse tumour xenograft model showed good tumour discrimination 24 h post i.v. injection of **1** with no observable toxicity.13*a* These positive *in vitro* and *in vivo* features are good indicators that bio-responsive NIR-AZA fluorophores warrant further investigation for translation towards clinical use in fluorescence-guided surgery. In recent preclinical tests, *always-on* NIR-AZA fluorophores have shown their potential for lymph node mapping and ureter identification using clinical instrumentation.^15^ In this report, we describe the synthesis, photophysical characterisation, *in vitro* and *in vivo* imaging assessment of bio-responsive NIR-AZA fluorophores conjugated to cyclic-RGD peptide sequences and polyethylene glycol polymer acting as active or passive targeting agents respectively.

Breast cancer is a key health concern for women, with over two million new cases diagnosed worldwide annually. Screening programs have resulted in most breast cancers being identified in the early stages with over 80% of breast cancer patients undergoing surgery as part of their treatment. Numerous trials have shown that for patients with between zero and three node metastases, breast-conserving surgery has similar or superior outcomes to mastectomy.^16^ As tumour-free surgical margins are critical to the success of breast-conserving surgery, utilising fluorescence guidance to improve surgical outcomes could have significant patient benefit.

Integrins are membrane bound cell adhesion receptors important for cell–cell and cell-extracellular matrix (ECM) interactions. They act as transmembrane linkers between extracellular ligands such as ECM proteins, growth factors, matrix degrading proteins and the cytoskeleton, which serves to modulate various essential signalling pathways in most cells.^17^ Integrins such as αvβ3 and αvβ5 (among others) are known to play a key role in tumour angiogenesis and are associated with the metastasis of solid tumours.^18^ Of the integrins, αvβ3 is one of the most studied as it is the most prevalent integrin involved in the regulation of angiogenesis and is widely expressed on tumour blood vessels.^19^ Over-expression of αvβ3 integrin has been associated with increased tumour growth in breast cancer and it has been shown that the activation of αvβ3 is a contributing factor for metastasis in breast cancer models.^20^ The tripeptide arginine–glycine–aspartic acid (RGD) sequence can recognise and bind ανβ3 and αvβ5 integrins and promote cellular internalisation with conjugates of the more stable cyclic variant c(RGDfK) being widely investigated as a selectivity enhancer for tumour therapies and diagnostics.^21^ The related iRGD peptide sequence (cCRGDKGPDC) has been reported to provide both specific integrin targeting and increased tumour uptake and penetration. It contains the RGD motif, which mediates binding to the endothelial cell membrane expressing the αv integrins but upon proteolytic cleavage a second neuropilin-1 (NRP-1) binding motif (CRGDK) is revealed to promote internalisation.^22^ Both RGD and iRGD conjugates have been investigated to improve drug selectivity with chemotherapeutic conjugates such as RGD-doxorubicin and RGD-paclitaxel showing promising preclinical results in breast carcinoma mouse models.^23^ RGD conjugates for fluorescent imaging using *always-on* probes has been explored in several preclinical models including breast cancers.^24^

For this study we have chosen one cRGD (c[RGDfK(PEG-PEG)]) and one iRGD (cCRGDKGPDC) peptide sequence for conjugation to the bio-responsive NIR-AZA imaging platform. It was hoped that these low molecular weight peptides would promote rapid uptake and switch-on of emission preferentially within tumours allowing a high tumour to background ratio (TBR) to be established without waiting for prolonged clearance times. In practice, it is envisaged that they would be administered and visualised intraoperatively, thereby not impeding the normal surgical or hospital workflow.

## Results and discussion

Incomplete tumour removal during surgical resection is closely related to cancer reoccurrence and patient survival rates. A major challenge in achieving cancer free margins is to fully distinguish between all of the cancerous growth and normal tissue during surgery. While high definition images obtained by PET, CT or MRI scans identify and diagnose tumour growths prior to surgery, such images are not overly useful to guide surgical resection during the operation. Currently, tumour margins are typically assessed by visual assessment and palpation of the tumour intraoperatively. However, the possibility of micro-invasion of the surrounding tissues can make it difficult to determine an adequate tumour-free excision margin. In this report, we have developed synthetic routes to RGD conjugated bio-responsive fluorophores, examined their photophysical and *in vitro* cellular emission profiles and tested their *in vivo* tumour imaging performance using a human breast tumour model in mice.

### Synthesis and characterisation

**Fig. 3:**
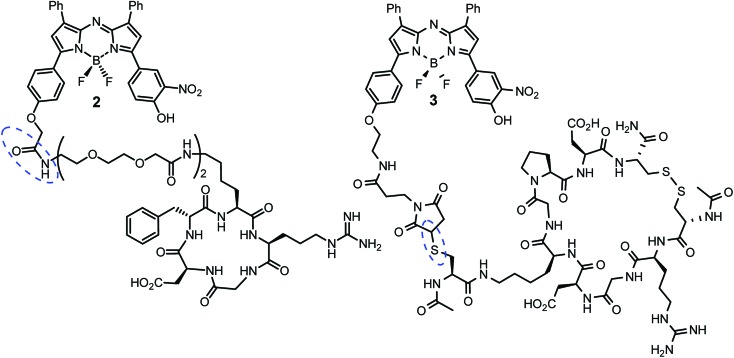
cRGD and iRGD bio-responsive NIR-AZAs **2** and **3**. Conjugation linkages highlighted in blue.

Cell uptake responsive probes **2** and **3** were selected for synthesis using activated ester/amine coupling to conjugate the cRGD sequence and cysteine to maleimide addition for the covalent linkage of the iRGD peptide sequence (Fig. 3). Two bio-conjugation approaches were adopted to confirm synthetic flexibility of NIR-AZA bio-fluorophores to functionalisation with targeting moieties.

**Scheme 1:**
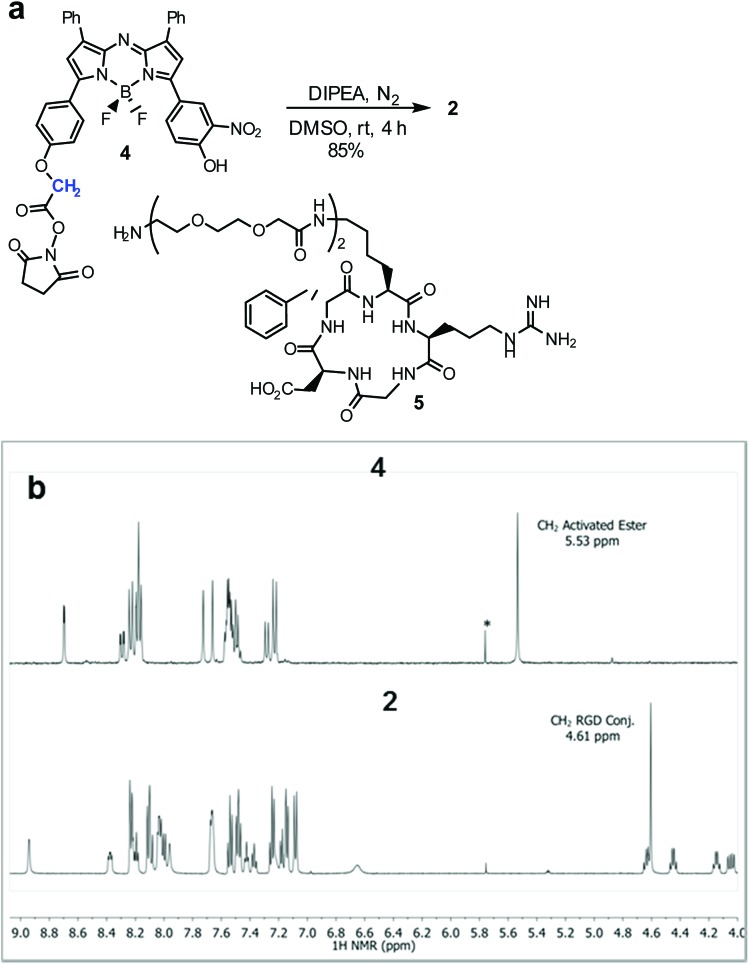
Synthesis of bio-responsive cRGD NIR-AZA conjugate **2**. (a) Conditions for activated ester/amine coupling of **4** and **5**. (b) ^1^H NMR spectra of **4** and **2** with key methylene peaks (shown in blue) indicated (*CH_2_Cl_2_).

Synthesis of **2** required fluorochrome **4** which has been previously reported, though only in reaction with an amino-pegylated polymer to produce **1** (Scheme 1).13*a* For this study, the amino-pegylated substituted cRGD substrate **5** (cyclo[Arg-Gly-Asp-D-Phe-Lys(PEG-PEG-NH_2_)]) was selected as it is known to be an ideal construct for housing the integrin recognising tripeptide sequence (Scheme 1a). The reaction of **4** and **5** in DMSO at rt was followed by HPLC and ^1^H NMR which showed a clean conversion to conjugate **2** in 4 h. Transformation of activated ester **4** into conjugate **2** was clearly distinguishable by the shift of the key methylene ^1^H NMR peak from 5.53 to 4.61 ppm (Scheme 1b). Purification of product was achieved using preparative reverse phase HPLC and the structure confirmed by high-resolution MS and NMR methods.

**Scheme 2:**
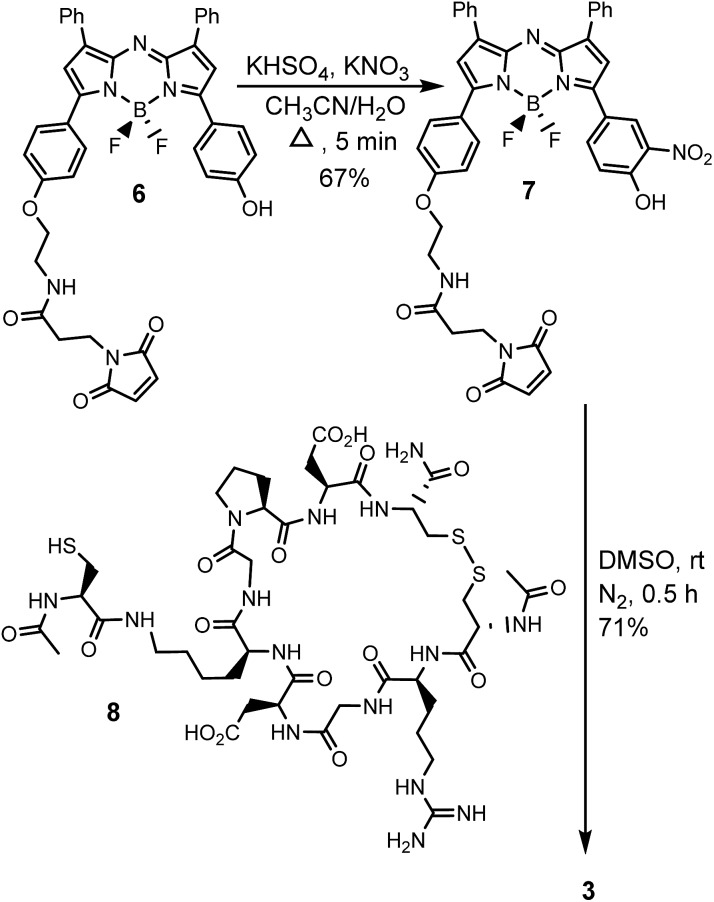
Synthetic route to bio-responsive iRGD NIR-AZA conjugate **3**.

The generation of iRGD conjugate **3** first required the synthesis of the corresponding maleimide-substituted fluorochrome **7** (Scheme 2). This was readily achievable starting from the previously reported derivative **6** which was subjected to the nitration conditions of KHSO_4_/KNO_3_ at reflux in CH_3_CN/H_2_O to yield the *o*-nitro phenol substituted substrate **7**.^25^ Synthesis of iRGD peptide **8** followed literature procedures to produce (cCRGDKGPDC) which was coupled with *N*-acetyl protected cysteine, through the amine of the lysine residue, to provide the final thiol substituted peptide.^26^ Bio-conjugation *via* cysteine to maleimide addition was efficiently achieved by reaction of **7** with iRGD **8** in DMSO at rt for 30 min to produce the required derivative **3** (Scheme 2). Product purification utilised preparative reverse phase HPLC with the structure confirmed by usual analytical methods (ESI†).

**Scheme 3:**
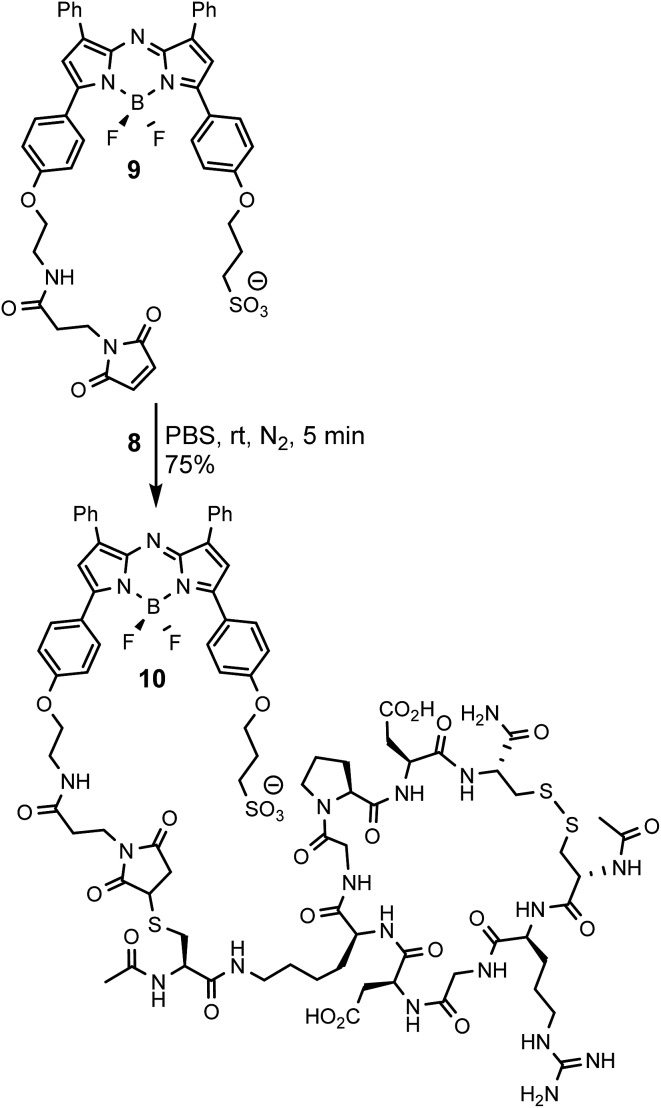
Synthesis of iRGD conjugated *always-on* NIR-AZA control **10**.

To allow comparisons be made between bio-responsive and non-responsive conjugates the iRGD substituted *always-on* control **10** was synthesised. Conjugate **10** is similar in structure to **3** but has the *o*-nitro phenol fluorescence switching substituent replaced by a water solubilizing alkylsulfonic acid group (Scheme 3). The synthetic route adopted to make this control utilised the reaction of known fluorochrome **9** with peptide **8** to produce **10**.^25^ The cysteine/maleimide coupling proceeded smoothly in phosphate buffered saline (PBS) at pH 7.2 with iRGD conjugated **10** obtained in good yield (Scheme 3, ESI†).

To test the extent of advantage gained from utilising integrin targeting RGD peptide conjugates such as **2** and **3***versus* a passive accumulating agent such as a PEG group, the pegylated bio-responsive **1** was also included for testing in the study as a comparative control (Fig. 2). This we envisaged would allow a direct imaging performance evaluation between bio-responsive fluorophores using either active targeting peptides or the passive enhanced permeability and retention (EPR) effect of a PEG group.

### Photophysical properties

The photophysical properties of the bio-responsive fluorophores **2** and **3** and the *always-on* control **10** were studied in solutions of Dulbecco's modified eagle's cell medium (DMEM) containing 10% fetal bovine serum (FBS). Absorption and emission wavelengths are listed in Table 1 and are, as would be expected for the NIR-AZA class, in the 690–730 nm range. At pH 7.4 fluorescence intensity of *always-on***10** was 17- and 15-fold greater than **2** and **3** respectively (Table 1). This illustrates both the emissive potential of the peptide conjugates and the ability to quench the bio-responsive derivatives in relevant biological media (Table 1, spectra and inset).

**Table 1:** Photophysical properties and emission spectra of **2**, **3**, and **10**^*a*^ ^,^^*b*^ ^,^^*c*^

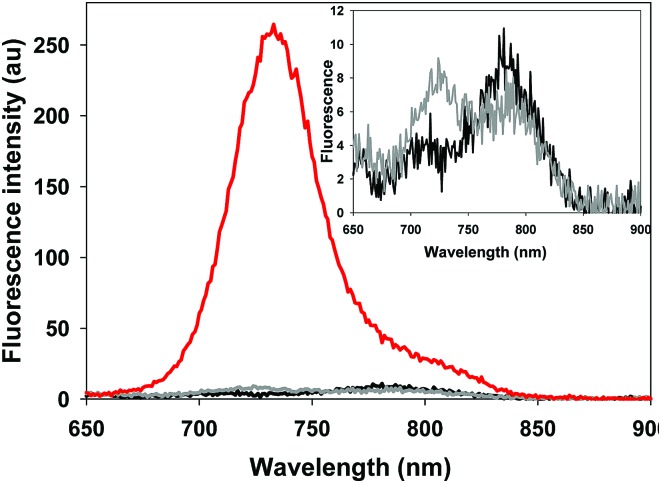				
Cpd	*λ*_max_ abs (nm) pH 7.2	*λ*_max_ abs (nm) pH 4.5	*λ*_max_ flu (nm)	FEF pH 7.4/4.5^*d*^ ^,^^*e*^
**1**	749	685	707	21 13*a*
**2**	755	693	713	23
**3**	757	696	716	18
**10**	705	703	733	1.0

^*a*^DMEM/10% FBS solutions with 4 mM TX-100.

^*b*^To allow a common comparison for each fluorophore solution across a wide acidity range 4 μM Triton X 100 was included in each solution.

^*c*^Fluorescence spectra taken at 5 μM concentration, **2** (black spectra) **3** (grey spectra) **10** (red spectrum), inset shows expansion of spectra for **2** and **3**.

^*d*^Fluorescence enhancement factor (FEF).

^*e*^Fluorescence quantum yields at pH 7.4 of **2** and **3** = 0.16 and 0.18 respectively (ESI).

**Fig. 4:**
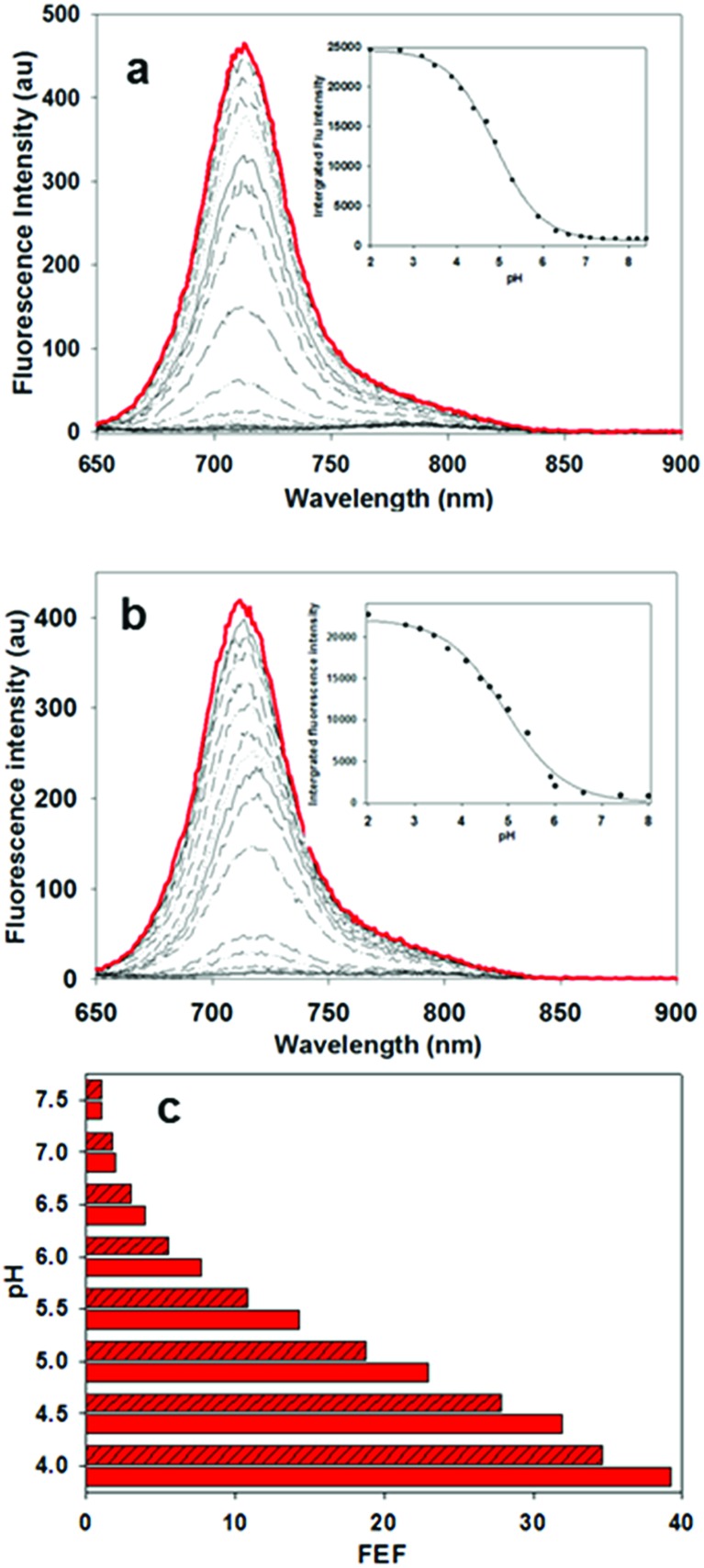
Responsive emission characteristics of **2** and **3** (5 μM) in DMEM/10% FBS solutions containing TX100. (a) Fluorescence spectra of **2** at pH ranging from 8.0 (black) to 2.0 (red); inset sigmoidal plot fit of integrated fluorescence intensity *versus* pH. (b) Fluorescence spectra of **3** at pH ranging from 8.0 (black) to 2.0 (red); inset sigmoidal plot fit of integrated fluorescence intensity *versus* pH. (c) Diagram representing FEF values from differing pH solutions of **2** (solid red bars) and **3** (crossed red bars).

In order to display the responsive nature and full emissive potential of **2** and **3**, their DMEM solutions were sequentially acidified (Fig. 4a and b). This caused a successive increase in emission intensity as acidity increased, with a maximum intensity reached at approximately pH 4. Plotting the measured data revealed p*K*_a_ values of 4.9 for both **2** and **3** which is consistent with the previously reported value of 4.6 for **1** (Fig. 4 insets, Fig. S1–S3†).13*a* This shows that the conjugating group does not overly influence the important *p*-nitro phenol emission-controlling feature. While it is recognized that the extracellular matrix of a solid tumour can be more acidic than normal tissue, intracellular organelles such as late endosomes and lysosomes are also acidic ranging between pH 4.5 and 5.5.^27^ Encouragingly, the measured fluorescence enhancement factor (FEF) for **2** and **3** between pH 7.2 and that of 4.5 (as found in lysosomes) was 23 and 18 respectively (Fig. 4c). As such, a switch-on of emission could be expected to occur both in the localised extracellular tumour microenvironment and upon cancer cell uptake. As emission quenching in the off states of **2** and **3** at pH 7.2 is highly effective, good background to noise differentials could be anticipated. In contrast, control fluorophore **10** showed no absorption or emission spectral changes between pH 7.2 and 4.5 (Fig. S4†).

The next stage involved testing **2**, **3** and control **10** in live cell imaging using the epithelial human breast cancer cell line MDA-MB 231 that is known to express membrane integrins including αvβ3 and αvβ5. MDA-MB 231 is a highly aggressive triple-negative cell line, with its invasiveness mediated by proteolytic degradation of the extracellular matrix.^28^ The metastatic invasive nature of MDA-MB 231 cells is closely associated with large acidic vesicles (LAV) in which endocytosed extracellular matrix can be digested by activated lysosomal proteinases such as cathepsin.^29^ As these intracellular LAVs have a pH of approximately 4, they also could activate the bio-responsive fluorophores upon cancer cell uptake in addition to lysosomes.

### *In vitro* live MDA-MB 231 cell imaging

**Fig. 5:**
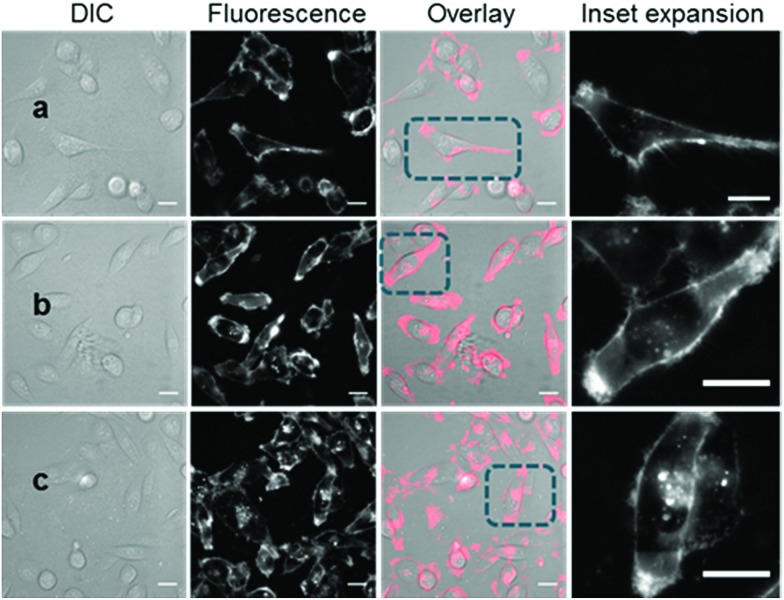
DIC and fluorescence microscopy imaging of live MDA-MB 231 cells over time following treatment with **10** (5 μM). Images taken at (a) 15 min (b) 1 h and (c) 2 h. Scale bars = 20 μm.

With the responsive nature of **2** and **3** established, the potential for translation of these constructs to real-time live cell imaging was investigated. In order to illustrate the imaging effect of the bio-responsive characteristics of **2** and **3** the *always-on***10** was also imaged as a positive control. For live MDA-MB 231 cell experiments, chamber slide seeded cells were placed in a widefield microscope surrounded by an incubator to maintain the temperature at 37 °C and CO_2_ at 5%, following which an imaging field of view containing viable cells was chosen. The cells were treated with either **2**, **3** or **10** (1–5 μM) and time-lapse NIR-fluorescence and differential interference contrast (DIC) images were acquired over 120 min. Image data showed that for *always-on***10**, a fluorescence specific to the plasma membrane was rapidly observed within 15 min, which could be attributed to its strong association with the cell membrane (Fig. 5a). As expected, the endothelial-like morphology of the cell line is distinguishable by its membrane filopodia projections which is characteristic of its metastatic invasive phenotype (Movie S1†). After 60 min incubation, both cell membrane and intracellular vesicle staining of the cytoplasm could be observed, both of which persisted at 120 min (Fig. 5b and c). A wider field of view showing a larger number of cells and Z-stack images can be seen in Fig. S5, S6 and Movie S2.†

**Fig. 6:**
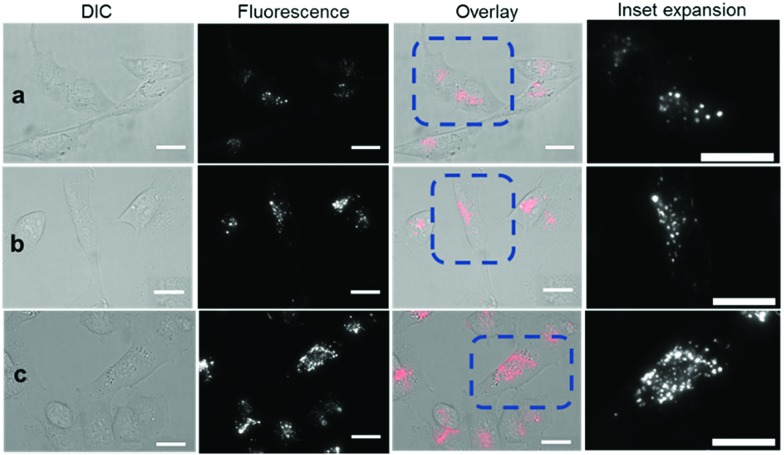
DIC and NIR fluorescence microscopy imaging of live MDA-MB 231 over time following incubation with **2** (5 μM). Images taken at (a) 15 min (b) 1 h and (c) 2 h. Scale bars = 20 μm.

Revealingly, the bio-responsive NIR-AZAs **2** showed no cell membrane staining in the first 15 min of incubation and only following this time point could intracellular regions of fluorescence be observed (Fig. 6a). The intracellular punctate staining pattern is consistent with those previously observed for **1** and are due to a selective bio-responsive switch-on of emission within the acidic vesicles of the cytoplasm.13a,^14^ The images revealed two distinct vesicle sizes, the smaller of which are attributable to cellular lysosomes and the bigger LAVs specific to the metastatic nature of MDA-MB 231 cells (Movie S3 and S4†). At 60 and 120 min the intracellular fluorescence intensity increased, but at no point was membrane fluorescence observed (Fig. 6b, c and Movie S5†). This lack of plasma membrane fluorescence shows that **2** can translocate across the membrane without fluorescence being activated. A wider field of view showing a larger number of cells can be seen in Fig. S7.† Z-Stack analysis of cells confirmed that regions of fluorescence were within the cytoplasm (Fig. S8 and Movie S6†). Similar results were obtained from imaging experiments using bio-responsive iRGD NIR-AZA **3** which can be seen in Fig. S9, S10 and Movie S7.† In addition, similar results were obtained from imaging experiments with HeLa Kyoto cells using bio-responsive **2** which can be seen in Movie S8.†

The different cell staining patterns between **10** and **2**, **3** over time shows the fidelity of the fluorescence switching and the potential signal to background contrast advantage of the bio-responsive NIR-AZA probes. The next challenge of this work was to examine if a preferential *in vivo* switch-on of bio-responsive NIR-AZAs in cancerous tumour could allow both early (due to initial switch-on) and later (due to retention of switched on probe) stage discrimination of tumour from background.

### *In vivo* tumour imaging

For this study, the human breast cancer cell line MDA-MB 231 was selected for its relevance to clinical forms of aggressive breast cancers for which the first line of treatment is often surgical resection. The ability of bio-responsive conjugates **1**, **2** and **3** were tested using subcutaneous tumours grown in nude mice. Fluorophore **10** was also included as a positive control in the study to demonstrate the advantage of using off to on responsive fluorescence over a constant emission. It was anticipated that **1**, **2** and **3** would remain predominately fluorescent silent until tumour uptake occurred causing a fluorescence signal modulation to on. Experimental measurement of changes in tumor-to-background ratio (TBR) over time was the preferred method to quantify differences between bio-responsive and *always-on* fluorophores. Pegylated bio-responsive **1** was included to compare the turn-on time differences between passive PEG and the active integrin targeting of **2** and **3**. The expectation being that the larger EPR dependent PEG conjugate would be slower.

Each fluorophore was subjected to *in vivo* analysis using a standard dosing set at 2 mg kg^–1^ delivered by i.v. tail vein injection. Post injection, images were acquired initially at regular intervals between 10 min and 9 h and thereafter less frequently at 24, 48 and 96 h. The method used for image analysis was consistent across all experiments with TBR values calculated by measuring tumour ROI fluorescence against an average of three equally sized background ROI regions, two of which were close to and one distant from the tumour (Fig. S11†). In previously reported preclinical studies, when imaging through the skin, a TBR ratio of two was shown to be a clinically relevant threshold.24a,^30^ As such, we adopted this value as a point of reference to compare result from different fluorophores and different time points for individual fluorophores.

**Fig. 7:**
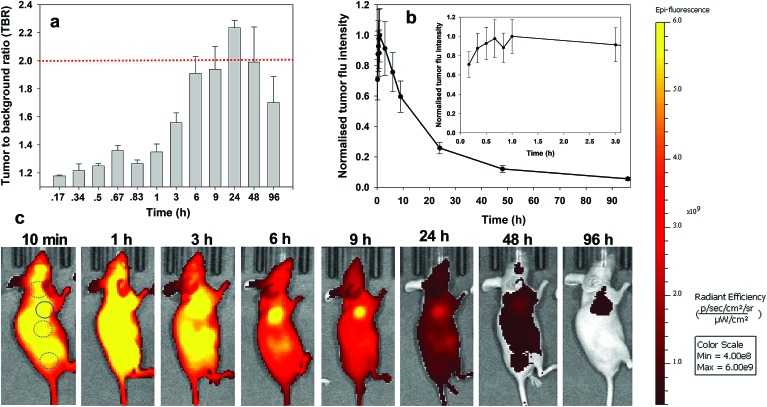
Analysis of fluorescence images for *always-on* iRDG NIR-AZA **10** (10.1 nmol) over time (*n* = 2). (a) Plot of TBR values at individual time-points over 96 h, dashed red line indicating threshold value of 2. Values determined by ROI total fluorescence signal of tumour divided by an averaged value of three independent background regions as measured by living image software v4.7. (b) Plot of tumour fluorescence intensity over 96 h, inset shows expansion of 0–3 h. (c) Representative *in vivo* fluorescence images of **10** using a MDA-MB 231 subcutaneous tumour model at different time points. First image shows selected tumour ROI (solid circle) and three background ROIs (dashed circles).

For *always-on* iRGD control **10**, from 10 to 60 min post i.v. injection an immediate strong and non-specific fluorescence was observable throughout the animals, with no discernible bias for tumour as demonstrated by the measured TBRs of below 1.3 (Fig. 7a). The TBR value marginally improved over the following 2 h with a TBR value of 1.5 achieved at 3 h post administration. While it is likely that **10** has begun to accumulate at the tumour site, the cancerous ROI is not readily distinguishable from the background fluorescence (Fig. 7c, see Fig. S12 in ESI† for additional time point images). By the 6 h time point the TBR had improved further to 1.9, though, it was not until 24 h post administration that a TBR above 2 was obtained. By this time, the overall fluorescence intensity had dropped approximately 75% fold from its peak (Fig. 7b). The TBR value of 2 was maintained out to 48 h as the emission intensity further decreased (90% of peak), and by 96 h it had fallen below the threshold. This sequence of TBR values comes about due to an initial distribution through normal and cancerous tissues followed by a faster clearance of **10** from normal tissue with retention within cancerous tissue. The sequence of images shown in Fig. 7c illustrates the general challenge facing *always-on* fluorophores, regardless of whether they are substituted with cancer specific targeting agents or not. As the process of accumulation and clearance of fluorophore from normal and cancerous tissues are both dynamic processes, the success or failure of an *always-on* probe relies on identifying the time point at which uptake and clearance for the different tissue types are most divergent from each other. This poses significant challenges for their use in surgical oncological practice with respect to patient-to-patient variances and complex hospital scheduling.

**Fig. 8:**
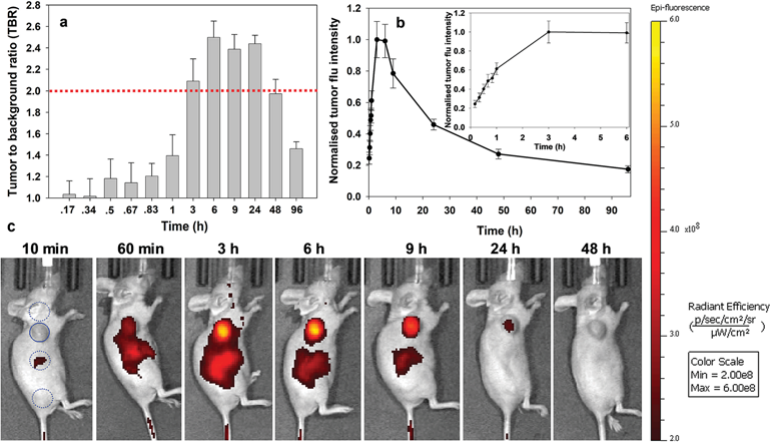
Analysis of *in vivo* fluorescence imaging for bio-responsive RGD NIR-AZA **2** (13.2 nmol) over time (*n* = 4). (a) Plot of TBR values at individual time-points over 96 h, dashed red line indicating threshold value of 2. Values determined by ROI total fluorescence signal of tumour divided by an averaged value of three independent background regions as measured by living image software v4.7. (b) Plot of tumour fluorescence intensity over 96 h, inset shows expansion of 0–3 h. (c) Representative *in vivo* fluorescence images of **2** using a MDA-MB 231 subcutaneous tumour model at different time points (scale bar identical to Fig. 9 and 10). First image shows selected tumour ROI (solid circle) and three background ROIs (dashed circles).

Analysis of the image timelines for bio-responsive RGD NIR-AZA **2** showed remarkable differences from control **10** (Fig. 8). As **2** is administered in solution at pH 7.2, it is non-fluorescent and remained virtually fluorescent silent within the vasculature immediately post injection ((Fig. 8b), see Fig. S13 in ESI† for additional time point images). At 60 min post injection, **2** begun to accumulate at the tumour region and the fluorescent signal had turned on giving a measured TBR of 1.4, with some background also observed from the adjacent liver (Fig. 8a and c). By 3 h a significant 4.1-fold increase in tumour fluorescence intensity had occurred along with a jump in TBR, surpassing the threshold of 2. The TBR value (2.5) reached a maximum at 6 h and maintained this level until 24 h. Importantly, emission intensity from the tumour reached a maximum within 3 h and there was no reduction in intensity between 3 and 6 h coinciding with when the TBR was at its maximum (Fig. 8b and inset). Encouragingly, even at 9 h only a ∼20% intensity reduction had occurred which provides a wide time frame in which tumour visualisation could be achieved (Fig. 8a). The ability of **2** to effectively tumour stain shortly after administration can be judged by the sequence of images shown in Fig. 8c. This we view very positively as not only is the threshold reached quickly, it is maintained for a prolonged time. This fits well with a clinical surgical workflow whereby the contrast agent could be administered at the start of surgery with intraoperative tumour visualization possible.

**Fig. 9:**
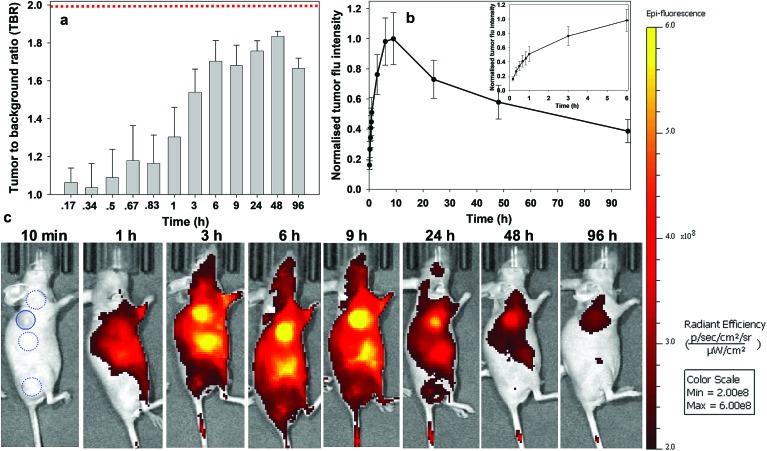
Analysis of *in vivo* fluorescence imaging for bio-responsive iRGD NIR-AZA **3** (10.5 nmol) over time (*n* = 4). (a) Plot of TBR values at individual time-points over 96 h, dashed red line indicating threshold value of 2. Values determined by ROI total fluorescence signal of tumour divided by an averaged value of three independent background regions as measured by living image software v4.7. (b) Plot of tumour (solid trace) and averaged background (dashed trace) fluorescence intensity over 96 h. (c) Representative *in vivo* fluorescence images of **3** using a MDA-MB 231 subcutaneous tumour model at different time points (scale bar identical to Fig. 8 and 10). First image shows selected tumour ROI (solid circle) and three background ROIs (dashed circles).

**Fig. 10 fig10:**
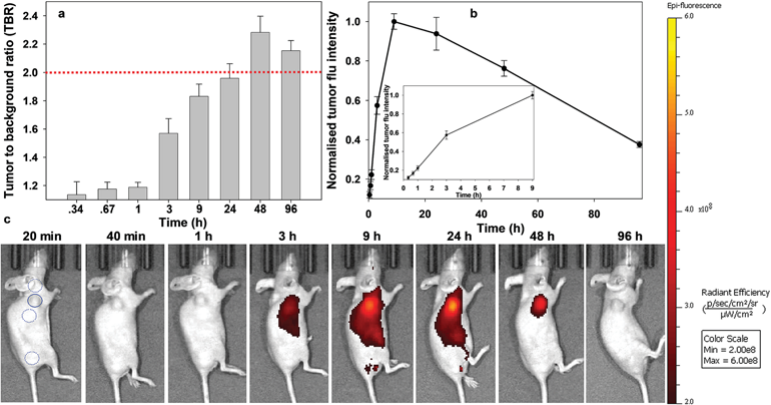
Analysis of *in vivo* fluorescence imaging for bio-responsive PEG NIR-AZA **1** (3.7 nmol) over time (*n* = 4). (a) Plot of TBR values at individual time-points over 96 h, dashed red line indicating threshold value of 2. Values determined by ROI total fluorescence signal of tumour divided by an averaged value of three independent background regions as measured by living image software v4.7. (b) Plot of tumour (solid trace) and averaged background (dashed trace) fluorescence intensity over 96 h. (c) Representative *in vivo* fluorescence images of **1** using a MDA-MB 231 subcutaneous tumour model at different time points (scale bar identical to Fig. 8 and 9). First image shows tumour ROI (solid circle) and three background ROIs (dashed circles).

Similarly, responsive iRGD NIR-AZA **3** also had emission suppressed at the start of imaging with very low fluorescence at 20 min and tumour accumulation evident at 60 min with a TBR of 1.4 (Fig. 9c [Fig fig10], see Fig. S14 in ESI† for additional time point images). Between 20 min and 3 h there was a 4.7-fold increase in tumour fluorescence intensity (Fig. 9b and inset). Yet, in comparison to **2** the extent of background signal was larger, though not brighter than the tumour itself and there was a longer delay until 6 h before the TBR threshold reached 1.7 (Fig. 9a and c). Disappointingly, the TBR never exceeded the threshold value of 2 with the best value of 1.8 recorded 48 h post injection.

Finally, the pegylated bio-responsive **1** was studied to determine the influence of the conjugating group on the time taken to reach maximum tumour fluorescence and TBR. Again, emission remained off in the beginning with a similar, but considerably time delayed, profile to that of **2**. TBRs remained low (∼1.2) for the first 60 min then rose to 1.6 and 1.8 at 6 and 9 h respectively. It took 9 h to reach maximum tumour fluorescence intensity with this level remaining relatively unchanged at 24 h. Pleasingly, the TBR threshold of 2 was reached at 24 h and this was maintained for a further 48 h. Comparing the changing TBRs of pegylated **1** and RGD **2** over time illustrates that substituting with the peptide sequence provides a considerably faster tumour accumulation. If clinically adopted, the slower time frame of **1** would most likely require its administration 24 h before surgery, though its long retention within the tumour may provide a prolonged window in which it could be imaged.

**Fig. 11:**
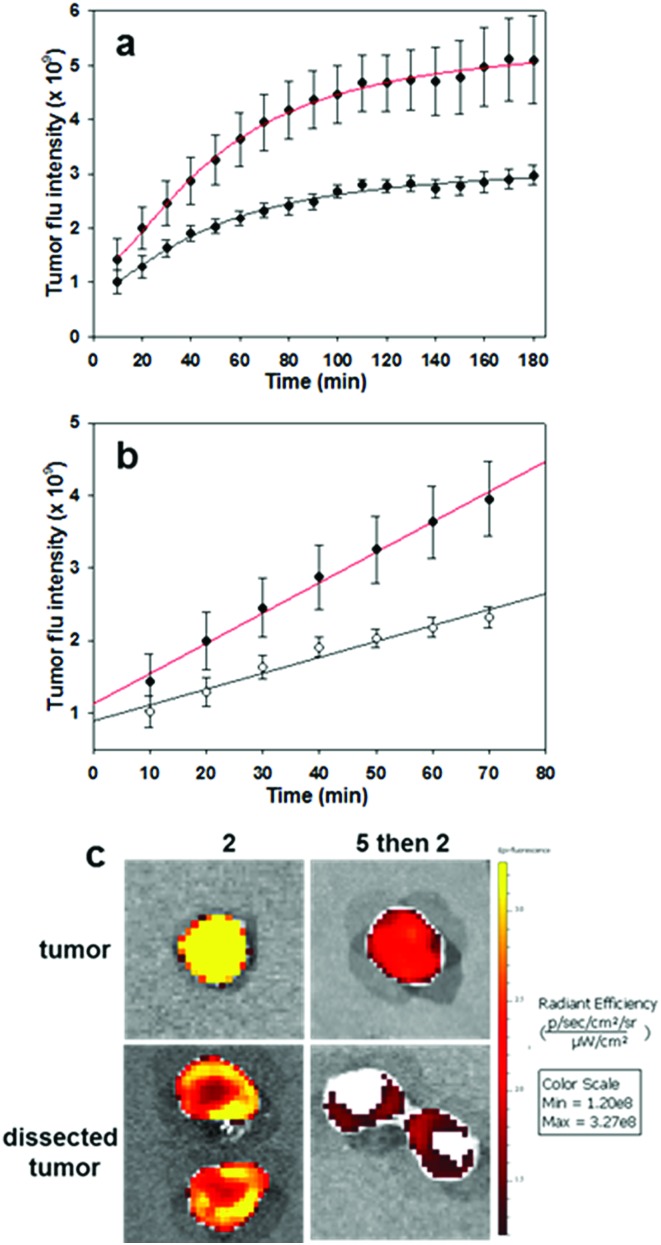
Competitive bind studies using RGD **5** and RGD NIR-AZA **2** (*n* = 4 pairs). (a) Plots of increasing tumour intensity over 3 h post administration for **2** (red trace) and for **5** followed by **2** (black trace). (b) Plots showing rate of increasing fluorescence for first 60 min post administration for **2** (red trace) and for **5** followed by **2** (black trace). (c) Imaging of resected tumours before and after dissection from animals treated **2** and for **5** followed by **2**.

To complete this study, further *in vivo* tests were performed on the most promising derivative RGD NIR-AZA **2**. To gain insight into the initial rate of tumour fluorescence turn on, images were acquired every 10 min for 3 h immediately after introduction of **2**. To establish that the RGD substituent was influencing the rate of tumour uptake, competitive binding or blocking experiments were also carried out. Experimentally this was achieved by first administering an i.v. tail injection of the RGD peptide **5** (6.8 fold equivalence excess) and following a short time period (5 min) **2** was then administrated. It would be expected that the first administration of RGD **5** would result in the integrin receptors being bound by the free peptide such that when NIR-AZA **2** was next introduced there would be a reduced fluorophore uptake and as such a lower switch-on of fluorescence. In order to make direct comparisons, pairs of mice with closely matching sized tumours were selected for each experiment (*n* = 4 pairs). One animal was first given RGD **5** then both animals were administered with **2**, following which fluorescence images of both animals were taken every 10 min for the following 3 h. Averaged results from four experiment showed a 1.75 fold reduction in the total tumour fluorescence intensity after three hours for the mice which were first treated with the peptide **5** prior to receiving the NIR-AZA conjugate **2***versus* the mice which only received **2** (Fig. 11a).

Additionally, the rate of fluorescence turn on in the first 80 min within the tumour that was not exposed to free RGD peptide was 1.9-fold higher than that exposed to the competing peptide (Fig. 11b). These results confirm that the peptide substituent of the RGD-fluorophore **2** is positively influencing tumour accumulation immediately following administration, which facilitates early time point imaging. Upon completion of imaging, tumours were resected from an animal pair with quantification of their fluorescence intensities showing a 3.9 fold suppression of emission intensity from the RGD peptide pre-treated animal (Fig. 11c and S15†). Encouragingly, dissection and imaging of the tumours showed that the fluorescence intensity was highest at the outer boundary of the tumour, which would be most beneficial for operative identification of the full extent of tumour margins (Fig. 11c). Finally, analysis of the fluorescence turn on profile of **2** alone showed that intensity had reached a near maximum at 80 min (Fig. 11a and b red traces). This indicates that **2** could be administered at the start of a surgical procedure with intraoperative tumour visualization taking place without significantly impeding the normal surgical workflow.

## Conclusion

In summary, three bio-responsive NIR-AZA fluorophore constructs have been synthesised conjugated to either active (RGD) or passive (PEG) tumour targeting groups and their photochemical, cellular and *in vivo* properties compared with an *always-on* fluorescent control. Each bio-responsive derivative showed excellent off to on fluorescence switching characteristics with large enhancement values. *In vitro* live MDA-MB 231 cell imaging experiments showed internal acidic organelle cell staining with the responsive probes **2** and **3**, contrasting with the *always-on* derivative **10** which first showed plasma membrane before internal organelle staining. This result proves that the fidelity of fluorescence switching is maintained in cellular experiments and is independent of the conjugating group.

A comprehensive *in vivo* assessment of tumour imaging performance for bio-responsive probes **1**, **2**, **3** and *always-on* derivative **10** was conducted with monitoring of the fluorescence distributions over 96 h following administration. As anticipated, the *always-on***10** gave an immediate, non-specific and very strong emission profile throughout animals whereas the bio-responsive **1**, **2** and **3** displayed relatively very low initial fluorescence. In the case of **10**, clearance from normal tissue with accumulation and retention in tumour, allowed for a TBR above 2 to be reached between 9 and 24 h. All three bio-responsive derivatives switched on within tumours at time points consistent with their conjugated targeting groups. cRGD **2** and iRGD **3** both had effective switch-on in the first hour though **2** had superior specificity for tumour than **3**. Probe **2** achieved the threshold TBR value of above 2 within 3 h and this was maintained for a further 24 h. Relatively, the PEGylated **1** had slower similar turn on characteristics taking 9 h to reach maximum fluorescence from the tumour. Despite the slower accumulation, its retention was biased to the tumour tissue with the threshold TBR value being reached at 24 h and maintained out to 96 h. The side-by-side imaging comparison of **1** and **2** is an important and unique illustration of the dynamic differences between passive EPR and active targeting in action.

Overall, the cRGD-conjugate **2** has been identified as showing excellent potential for clinical translation for intraoperative fluorescence guided tumour margin identification. Its bio-responsive nature with early accumulation at the tumour periphery may overcome the inherent drawback of *always-on* fluorophores requiring prolonged clearance times. The PEGylated derivative **1** does also offer potential for clinical translation though its slow switch-on rate may ultimately limit its clinical scope.

The next ongoing stage of this research is to record continuous NIR-fluorescence video of the bio-responsive turn on at tumour margins to gather more kinetic data on the tissue dependent rates of emission increase over the first 90 min. This real-time data will be utilised in conjunction with specifically developed algorithms for dynamic image analysis that could provide the surgical team with an augmented reality (AR) heat map representation of the tissue to be excised during the operation. An intraoperative use of dynamic fluorescence tissue imagery combined with AI analysis and a clinical AR interface has the potential to transform surgical practice.

## Experimental section

Detailed experimental procedures and characterisations are provided in the ESI.†

## Author contributions

D. W. synthesised, measured photophysical properties and analysed compounds **3**, **7**, **9**, **10**, carried out *in vitro* cell imaging and *in vivo* image analysis. H. D. synthesised, measured photophysical properties and analysed compounds **1**, **2**. M. G. developed the synthetic route to compounds **1**, **2**, **4** and determined their photophysical properties. E. C. performed *in vivo* imaging studies, preliminary analysis and ethical approval application with assistances from B. L. and W. M. G., R. E. synthesised and analysed compound **8**. D. F. O. conceived the project, designed experiments and wrote the manuscript with input from all the co-authors.

## Conflicts of interest

DOS declares the following competing financial interest. Patents have been filed on BF_2_-azadipyrromethene based NIR fluorophores (EP2493898 and US8907107) in which he has a financial interest.

## Supplementary Material

Supplementary information

Supplementary movie

Supplementary movie

Supplementary movie

Supplementary movie

Supplementary movie

Supplementary movie

Supplementary movie

Supplementary movie
