# Supplementary material for: RGD conjugated cell uptake off to on responsive NIR-AZA fluorophores: applications toward intraoperative fluorescence guided surgery
Source: Chem Sci. 2019 Jun 21;10(29):6944–56. doi: 10.1039/c9sc02197c (PMC6686729; doi:10.1039/c9sc02197c)
Supplement: Supplementary file 1 [file SC-010-C9SC02197C-s001.pdf]

Electronic Supplementary Material (ESI) for Chemical Science.
This journal is © The Royal Society of Chemistry 2019

Supporting Information

RGD Conjugated Cell Uptake Off to On Responsive NIR-AZA Fluorophores: Applications toward Intraoperative Fluorescence Guided Surgery†

Dan Wu,[1] Harrison C. Daly,[1] Marco Grossi,[1] Emer Conroy,[2] Bo Li,[2] William M. Gallagher,[2] Robert Elmes,[3] Donal F. O'Shea*[1]

[1] Department of Chemistry, RCSI, 123 St. Stephen's Green, Dublin 2, Ireland.

[2] School of Biomolecular and Biomedical Science, Conway Institute, University College Dublin, Belfield, Dublin 4, Ireland.

[3] Department of Chemistry and Maynooth University Human Health Institute, Maynooth University, Maynooth, Ireland.

Department of Chemistry, RCSI, 123 St. Stephen's Green, Dublin 2, Ireland.

E-mail: donalfoshea@rcsi.ie

Table of Contents

General methods S3

Fig. S1 Absorption and fluorescence pH titration of **1** **S6**

Fig. S2 Absorption pH titration and fluorescence spectra of **2** **S6**

Fig. S3 Absorption pH titration and fluorescence spectra of **3** **S7**

Fig. S4 Absorption and fluorescence pH titration of **10** **S7**

Fig. S5 DIC and fluorescence microscopy imaging of live MDA-MB 231 cells over time following treatment with **10** **S8**

Fig. S6 Z-Stack fluorescence microscopy images of **10** in MDA-MB 231 cells **S9**

Fig. S7 DIC and fluorescence microscopy imaging of live MDA-MB 231 cells over time following treatment with **2** **S10**

Fig. S8 Z-Stack fluorescence microscopy images of **2** in MDA-MB 231 cells **S11**

Fig. S9 DIC and fluorescence microscopy imaging of live MDA-MB 231 cells over time following treatment with **3** **S12**
Fig. S10 Z-Stack fluorescence microscopy images of **3** in MDA-MB 231 cells **S13**
Fig. S11 Example showing background selection for *in vivo* TBR study **S14**
Fig. S12 Additional time point images for Fig. 7 **S15**
Fig. S13 Additional time point images for Fig. 8 **S15**
Fig. S14 Additional time point images for Fig. 9 **S16**
Fig. S15 Excised tumour images of RGD competitive binding **S16**
Synthesis and analysis of compounds **2**, **3**, **7**, **10** **S17**
Analytical data spectra for **1**, **2**, **3**, **7, 10** **S23**
Movie S1 Membrane filopodia imaging with **10**.
Movie S2 Z-Stack fluorescence microscopy video with **10**.
Movie S3 Live video of large acidic vesicles (LAV) imaging with **10**.
Movie S4 Live video of large acidic vesicles (LAV) imaging with **2**.
Movie S5 Live imaging of **2** over 2 hours.
Movie S6 Z-Stack fluorescence microscopy video of **2**.
Movie S7 Z-Stack fluorescence microscopy video of **3**.
Movie S8 Z-Stack fluorescence microscopy video of **2** in Hela Kyoto cell line after 1 hr incubation.

Experimental

General Methods.

All reactions involving air-sensitive reagents were performed under nitrogen in oven-dried glassware using syringe-septum cap technique. All solvents were purified and degassed before use. Chromatographic separation was carried out under pressure on Merck silica gel 60 using flash-column techniques. Reactions were monitored by thin-layer chromatography (TLC) carried out on 0.25 mm silica gel coated aluminum plates (60 Merck F_{254}) using UV light (254 nm) as visualizing agent. Unless specified, all reagents were used as received without further purifications. ^{1}H NMR and ^{13}C NMR spectra were recorded at room temperature at 400 MHz or 500 MHz and 100 MHz respectively, and calibrated using residual non-deuterated solvent as an internal reference. ESI mass spectra were acquired using Advion Expression Mass Spectrometer in positive and negative modes as required. Advion Data Express v5.1 software were used to carry out the analysis. ESI (HRMS) mass spectra were acquired using a microTOF-Q spectrometer interfaced to a Dionex UltiMate 3000 LC in positive and negative modes as required. MicroTof control 3.2 and HyStar 3.2 software were used to carry out the analysis. The desalting purification was completed via size exclusion chromatography Sephadex G-25 (30 × 300 mm) and analyzed by reverse phase chromatography on a HPLC (Shimadzu) equipped with analytical (YMC-Triart Phenyl, 4.6 × 150 mm I.D. S-5 μm, 12 nm) columns. Analytical elute with acetonitrile and water. Combined pure fraction was dried by lyophilization. All absorbance spectra were recorded with a Varian Cary 50 Scan UV-visible spectrophotometer and fluorescence spectra were recorded with a Varian Cary Eclipse fluorescence spectrophotometer. Data was normalized in SigmaPlot 8, pKa values were generated from scatter plots of pH values on the x-axis and integrated fluorescent intensity values on the y-axis using the dynamic curve fit function. MDA-MB 231 cells obtained from Caliper Life Sciences.

Fluorescence response to pH variation

Separately, compounds **1**, **2**, **3**, **10** were accurately weighed (3 mg for **1**, 1 mg for **2**, **3**, **10**) and dissolved in PBS with desired volume to make stock solution (500 μM). Stock solutions were diluted to a concentration of 5 μM with DMEM supplemented with 10% FBS and TX-100 (4mM). The pH of stirred solutions were adjusted with diluted HCl or $NaHCO_3$ (0.1/0.5 M) to obtain a range from 8 to 2. At each pH value UV-vis absorption and fluorescence spectra were recorded. Excitation = 630 nm. Emission range = 650 – 900 nm; slit widths: 5/5 nm.

Cell culture and live-cell imaging

MDA-MB 231 human breast cancer cells were seeded on to an eight well chamber slide (Ibidi) at a density of 1 × 10^4 cells per well 24 h before imaging. Cells were cultured in Dulbeccos Modified Eagles Media supplemented (DMEM) with 10% fetal bovine serum (FBS), 1% L-Glutamine, and Penicillin-Streptomycin (1000 U/mL), and incubated at 37 °C and 5% CO_2. The slide was place on the microscope stage surrounded by an incubator to maintain the temperature at 37 °C and CO_2 at 5%. DIC imaging was used to choose a field of view and focus on a group of cells.

Fluorescence and DIC images were acquired on an Olympus IX73 epi-fluorescent microscope fitted with an Andor iXon Ultra 888 EMCCD and controlled by MetaMorph (v7.8). Fluorescence illumination was provided by a Lumencor Spectra X light engine containing a solid-state light source. NIR: excitation filter = 640 (14) nm. Emission filter = 705 (72) nm. Images were acquired using a 60×/1.42 oil PlanApo objective (Olympus). Image processing was completed by using software ImageJ 1.52n (National Institutes of Health, USA).

***In vivo* mouse imaging**

All in vivo experiments were conducted in University College Dublin, Ireland in compliance with relevant laws, and their institutional guidelines for animal protocols and the guidelines issued by the Ethical Committee of University College Dublin and under license from the Health Products Regulatory Authority (HPRA), Dublin, Ireland which approved the experiments.

All *in vivo* work was carried out in the biomedical facility, University College Dublin (UCD). MDA-MB-231, a human breast adenocarcinoma cell line, was obtained from Caliper Life Sciences. Cells were maintained as a mono-layer culture in Minimum Essential Medium containing 10% (v/v) fetal bovine serum and supplemented with 1% (v/v) L–glutamine, 50 U/mL penicillin, 50 μL/ mL streptomycin, 1% (v/v) sodium pyruvate and 1% (v/v) non-essential amino acids. All cells were maintained in 5% CO_2 (v/v) and 21% O_2 (v/v) at 37°C.

Balb/C nu/nu mice (Harlan) were housed in individually ventilated cages in temperature and humidity-controlled rooms with a 12 hours light dark cycle. 2-5 Million MDA-MB 231 cells in 100 μL of a DPBS:Matrigel (50:50) solution were injected subcutaneously behind the fore limb of the 5-week-old mice using a 25-g needle. Tumors reached an average diameter of 6 mm prior to imaging experiments.

Optical imaging was performed with an IVIS Spectrum small-animal in vivo imaging system (Caliper LS) with integrated isoflurane anesthesia. This system consists of a cooled slow scan CCD camera and a light tight chamber that facilitates detection of very low light levels. A non-injected control animal was included. Images were acquired at regular intervals post injection with initially frequent

images taken at early time points with less frequent imaging thereafter. Images were taken using the settings of excitation 675 nm (30 nm band pass filter) and emission 720 nm (20 nm band pass filter) narrow band pass filters and were analyzed using Living Image Software v4.7.2 (Caliper LS). The tumor-to-background ratio (TBR) was calculated by using the measured fluorescence signal in the ROIs of tumor target and average background.

$$\text{TBR} = \frac{\text{mean signal tumor}}{\text{mean signal background}}$$

Three different background ROIs of same area size were selected and signal averaged to give the mean signal background value (see Fig. S11).

The fluorescence intensity of the animal was measured and normalized to photons per second per centimeter squared per steradian per micro-Watt per centimeter squared [(p/sec/cm^2/sr) /(μW/cm^2)]. Mice were injected via tail vein (2 mg/kg) as follows; [n = 4 for **1** (3.7 nmol)], [n = 4 for **2** (13.2 nmol)], [n = 4 for **3** (10.5 nmol)], [n = 2 for **10** (10.1 nmol)], anesthetized, and imaged at various time points post injection. All fluorescence images were acquired with two second exposure (f/stop = 2). Color scale was adjusted and kept same for each experiment to show good contrast of target tumor region to background over time. The fluorescence intensity was not affected by color scale adjusting.

***In vivo* mouse RGD competitive binding study**

For RGD competitive binding study, mice injected with **2** (13.2 nmol) with or without pre-injection with RDG **5** (89.5 nmol) and were imaged at 10 min interval for 3 hours, and then immediately euthanized by inhalation of carbon dioxide. For quantitative comparison, regions of interests (ROIs) were drawn over tumor and the results presented as mean ± standard deviation (SD) for a group of four animals.

Fig. S1. Absorption and fluorescence pH titration of **1**

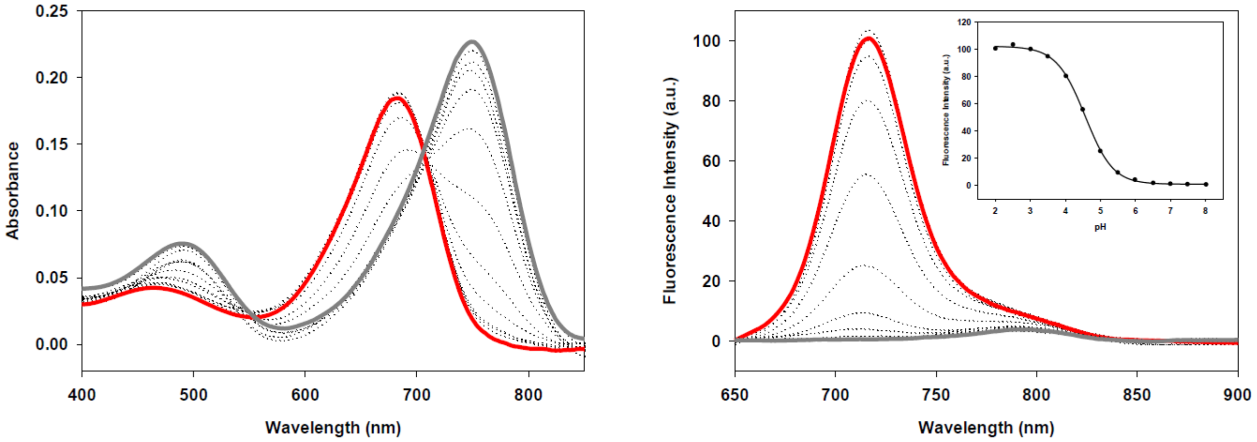

Fig. S1 Absorbance (left) and fluorescence (right) spectra of **1** (5 µM) in PBS buffer/TX-100 (4 mM) starting at pH 8 (grey line) to pH 2 (red line). Fluorescence excitation: 630 nm; range: 650- 900 nm; slit widths: 5/5. Inset: fluorescence intensity at λmax = 716 nm vs. pH; sigmoidal plot fit resulted in pKa = 4.6.

Fig. S2. Absorption pH titration and fluorescence spectra of **2**

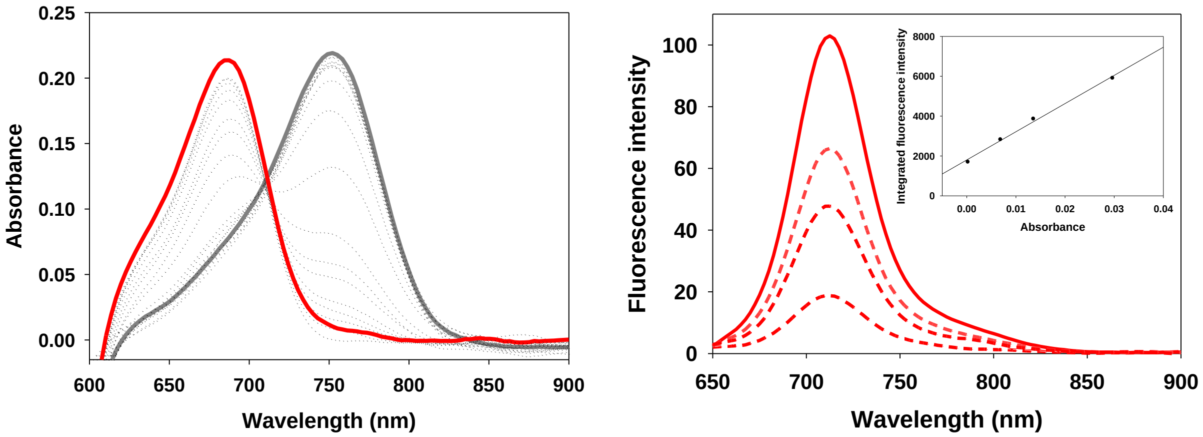

Fig. S2 Absorbance (left) spectra of **2** (5 µM) in DMEM (10% FBS) /TX100 (4 mM) starting at pH 8 (grey line) to pH 3 (red line). Fluorescence (right) spectra of series dilution (3 µM, 1.8 µM, 1.3 µM, 0.5 µM) in DMEM (10% FBS) /TX100 (4 mM) at pH=3. Fluorescence excitation: 630 nm; range: 650- 900 nm; slit widths: 5/5. Inset: Integrated fluorescence intensity vs. Absorbance; calculated fluorescence quantum yield Φ = 0.16.

Fig. S3. Absorption pH titration and fluorescence spectra of **3**

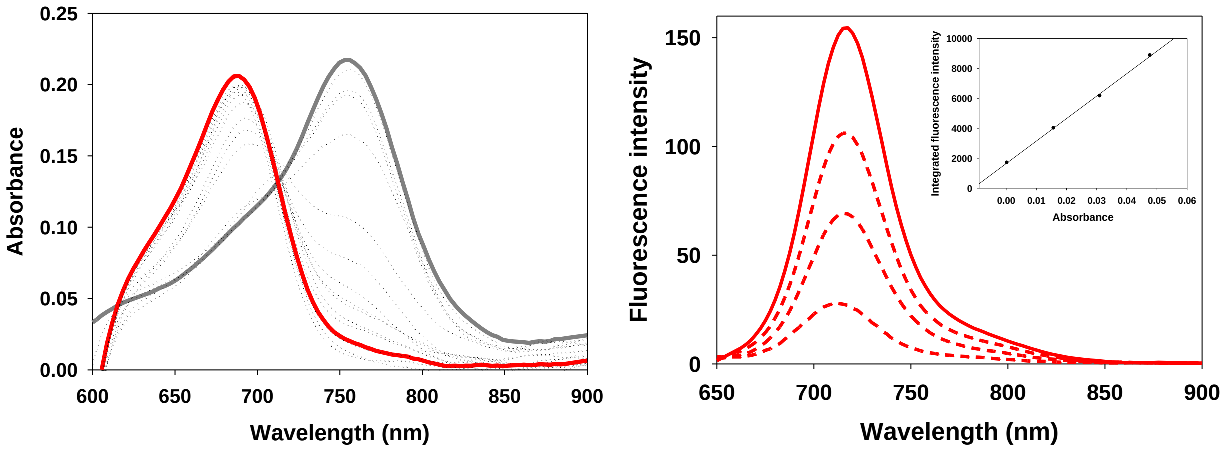

Fig. S3 Absorbance (left) spectra of **3** (5 µM) in DMEM (10% FBS) /TX100 (4 mM) starting at pH 8 (grey line) to pH 3 (red line). Fluorescence (right) spectra of series dilution (3.5 µM, 2.6 µM, 1.3 µM, 0.8 µM) in DMEM (10% FBS) /TX100 (4 mM) at pH=3. Fluorescence excitation: 630 nm; range: 650- 900 nm; slit widths: 5/5. Inset: Integrated fluorescence intensity vs. Absorbance; calculated fluorescence quantum yield Φ = 0.18.

Fig. S4. Absorption and fluorescence spectra of **10** at pH 7.4**,** 6.6**,** 5.6 and 3.

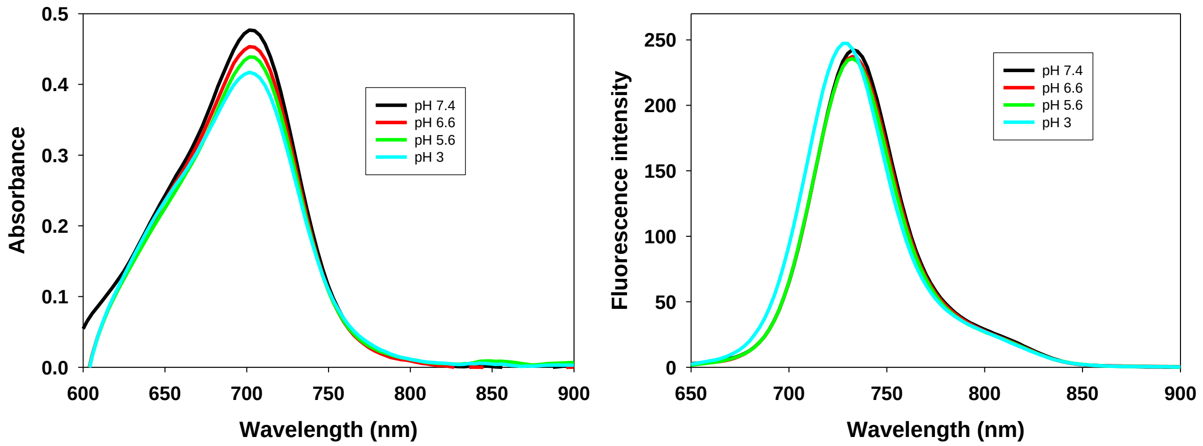

Fig. S4 Absorbance (left) and fluorescence (right) spectra of **10** (5 µM) in DMEM (10% FBS) /TX100 (4 mM). Fluorescence excitation: 630 nm; range: 650- 900 nm; slit widths: 5/5.

Fig. S5 DIC and fluorescence microscopy imaging of live MDA-MB 231 cells over time following treatment with **10**

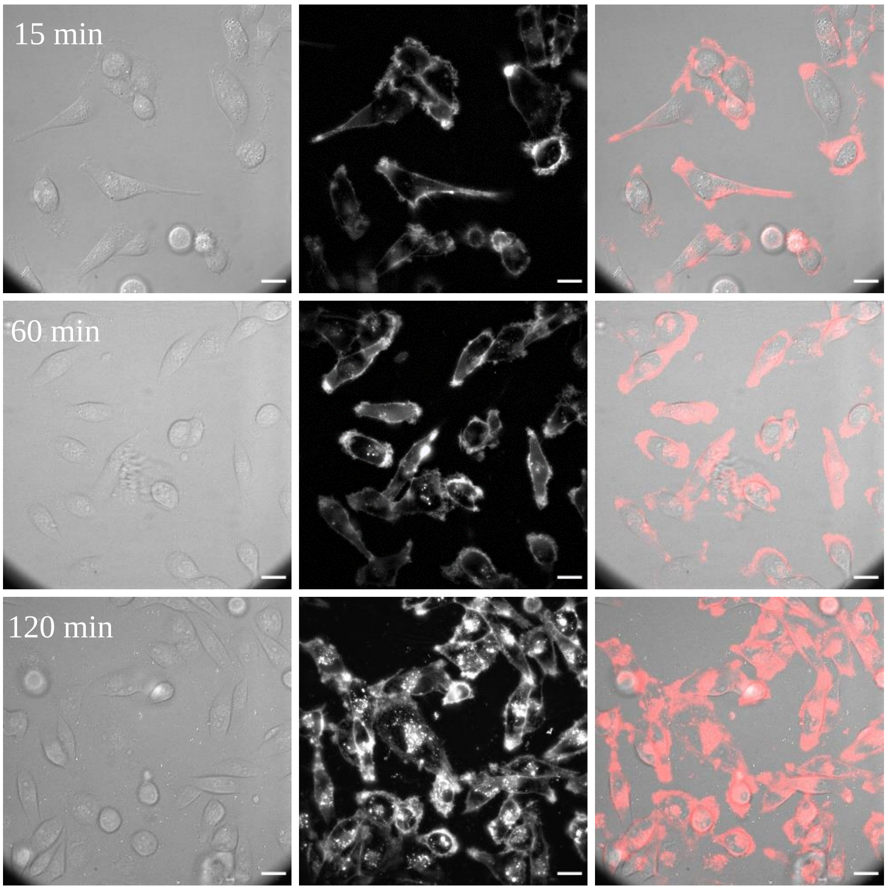

Fig. S5 Larger field of view of DIC and fluorescence microscopy imaging of live MDA-MB 231 cells over 2 h following treatment with **10** (5 µM). Scale bar = 20 µm.

Fig. S6 Z-Stack fluorescence microscopy images of **10** in MDA-MB 231 cells

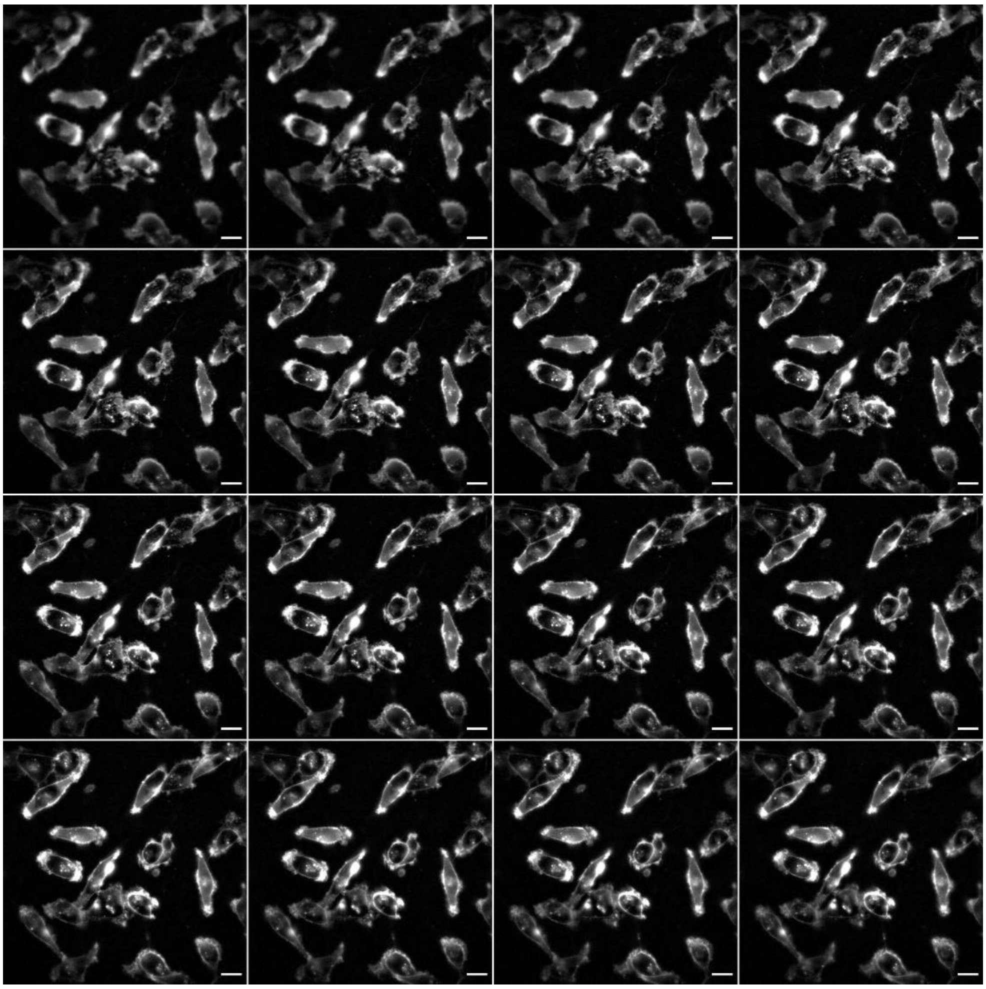

Fig. S6 Z-stack was acquired in live cells. Conc. of **10**, 5 µM. Scale bar = 20 µm. For montage see Movie S2.

Fig. S7 DIC and fluorescence microscopy imaging of live MDA-MB 231 cells over time following treatment with **2**

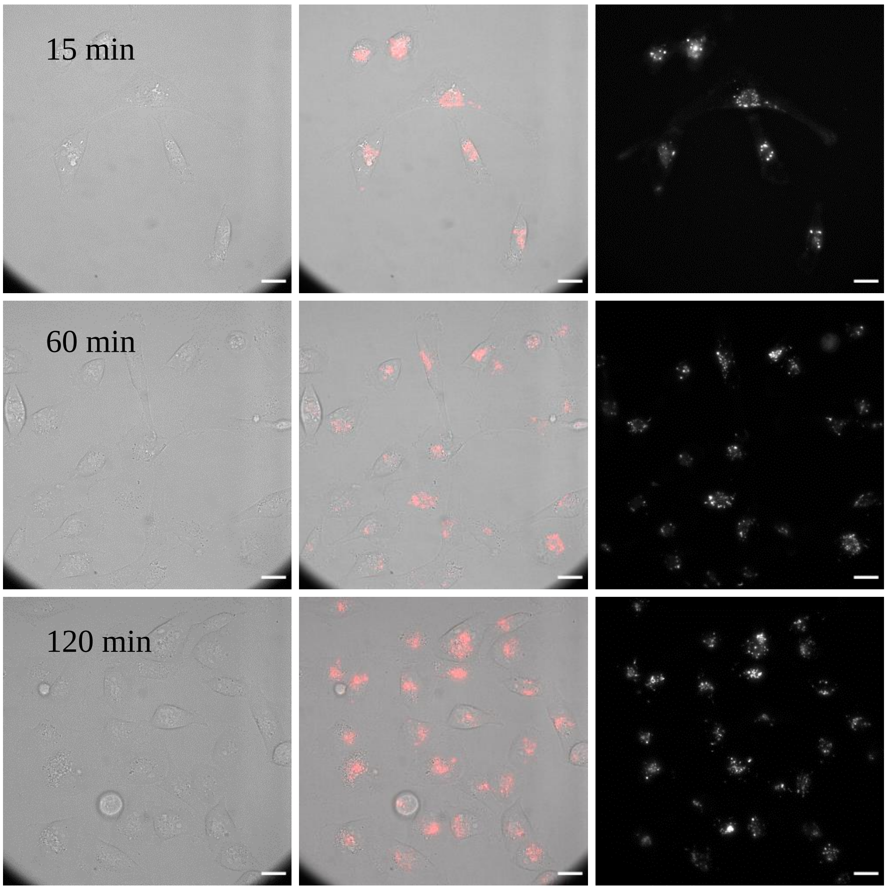

Fig. S7 Larger field of view of DIC and fluorescence microscopy imaging of live MDA-MB 231 cells over 2 h following treatment with **2** (5 µM). Scale bar = 20 µm.

Fig. S8 Z-Stack fluorescence microscopy images of **2** in MDA-MB 231 cells

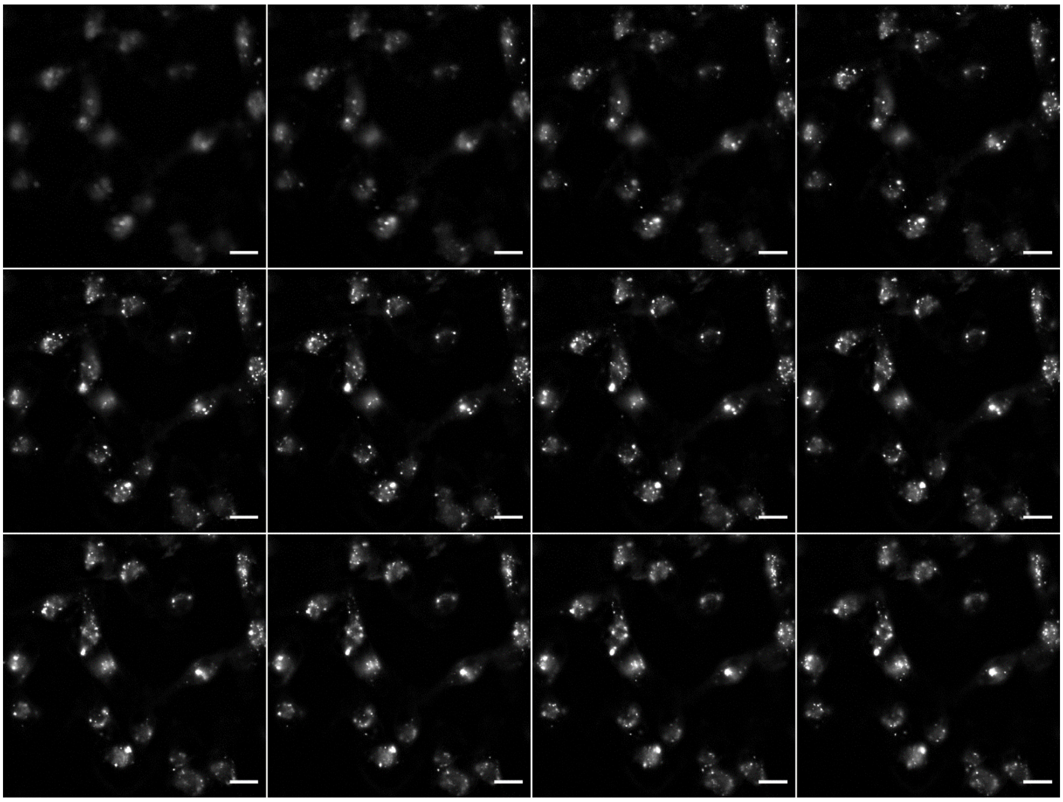

Fig. S8 Z-stack was acquired in live cells. Conc of **2**, 5 µM. Scale bar = 20 µm.
For montage see Movie S6.

Fig. S9 DIC and fluorescence microscopy imaging of live MDA-MB 231 cells over time following treatment with **3**

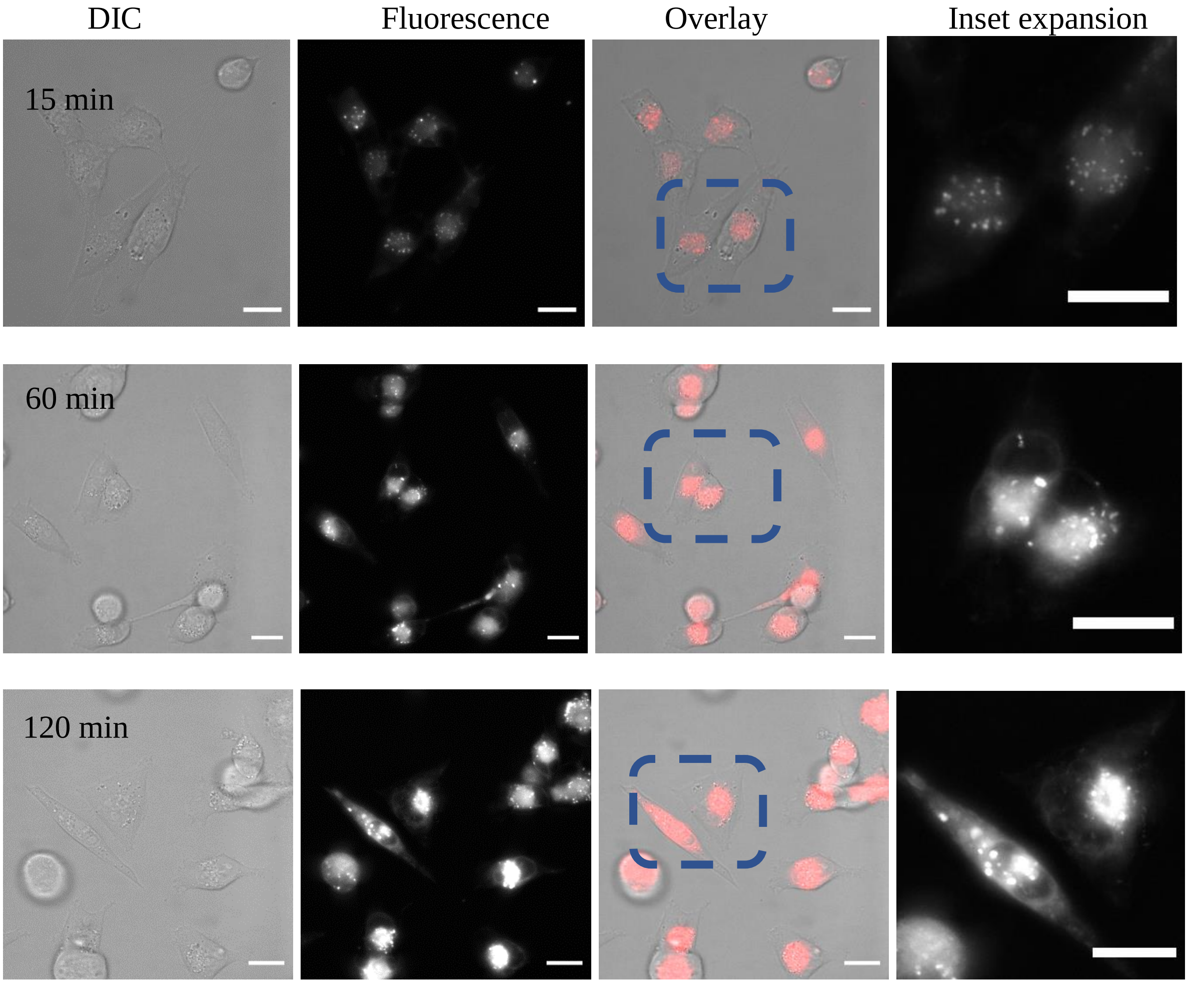

Fig. S9 DIC and fluorescence microscopy imaging of live MDA-MB 231 cells over 2 h following treatment with **3** (5 µM). Scale bar = 20 µm.

Fig. S10 Z-Stack fluorescence microscopy images of **3** in MDA-MB 231 cells

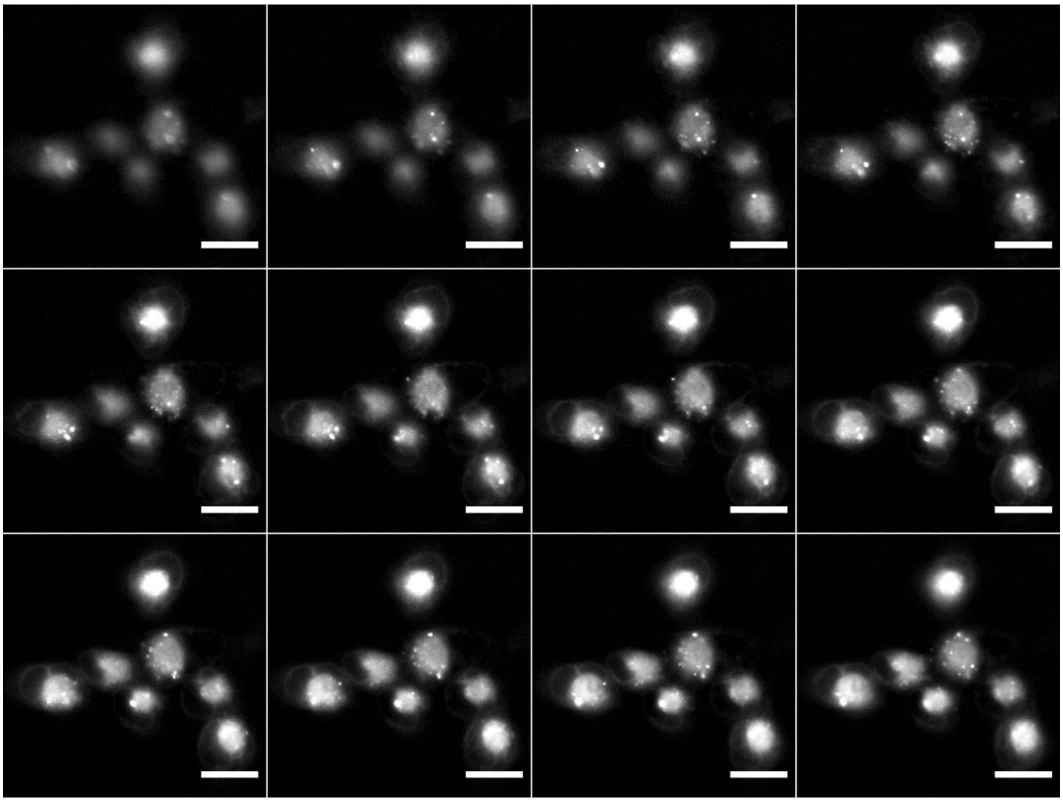

Fig. S10 Z-stack was acquired in live cells, conc. of **3**, 5 µM. Scale bar = 20 µm. For montage see Movie S7.

Fig. S11 Examples showing background selection for *in vivo* TBR study.

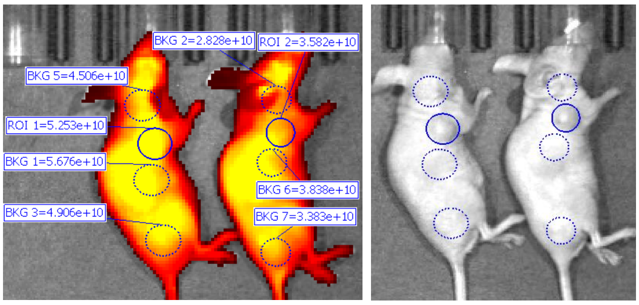

Representative example background selections for **10**, tumor ROI (solid circle), three different background ROI of same area size are selected (dashed circle). Left fluorescence image, right photograph image.

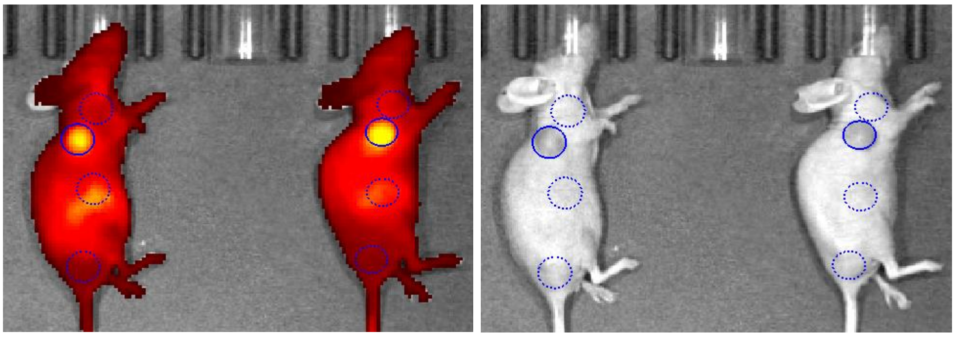

Representative example background selections for **2**, tumor ROI (solid circle), three different background ROI of same area size are selected (dashed circle). Left fluorescence image, right photograph image.

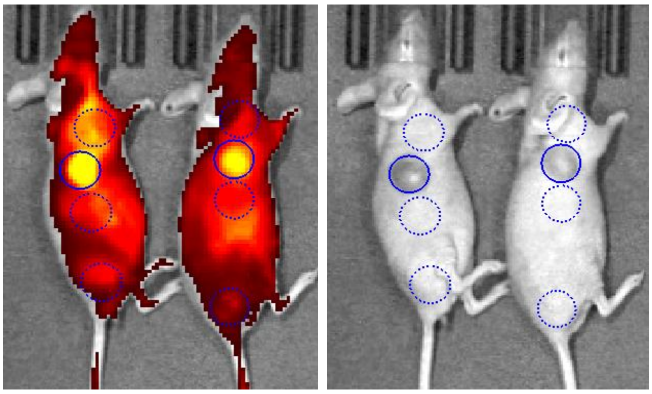

Representative example background selections for **3**, tumor ROI (solid circle), three different background ROI of same area size are selected (dashed circle). Left fluorescence image, right photograph image.

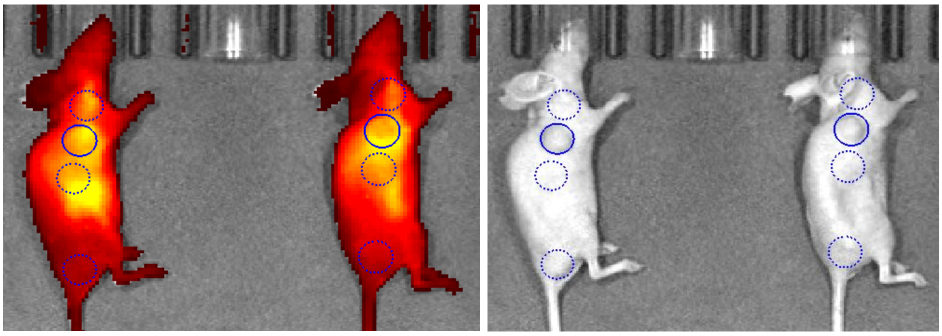

Representative example background selections for **1**, tumor ROI (solid circle), three different background ROI of same area size are selected (dashed circle). Left fluorescence image, right photograph image.

Fig. S12 Additional time point images of **10** for Fig. 7

20 min 30 min 40 min 50 min

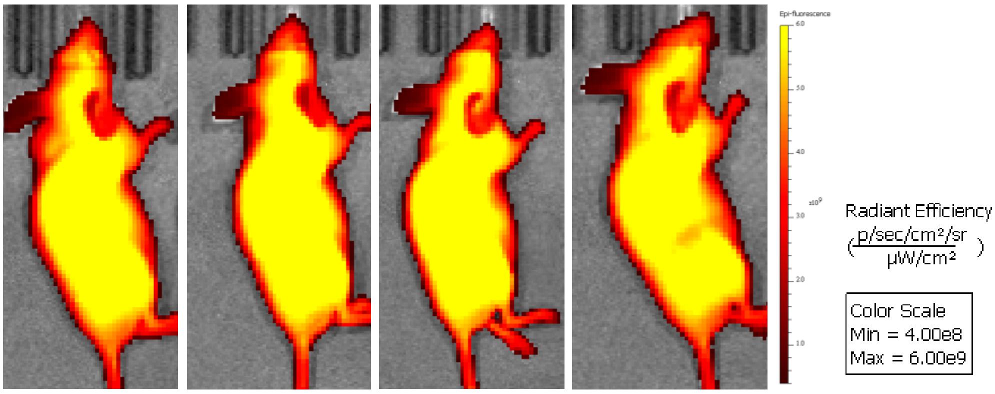

Fig. S13 Additional time point images of **2** for Fig. 8.

20 min 30 min 40 min 50 min

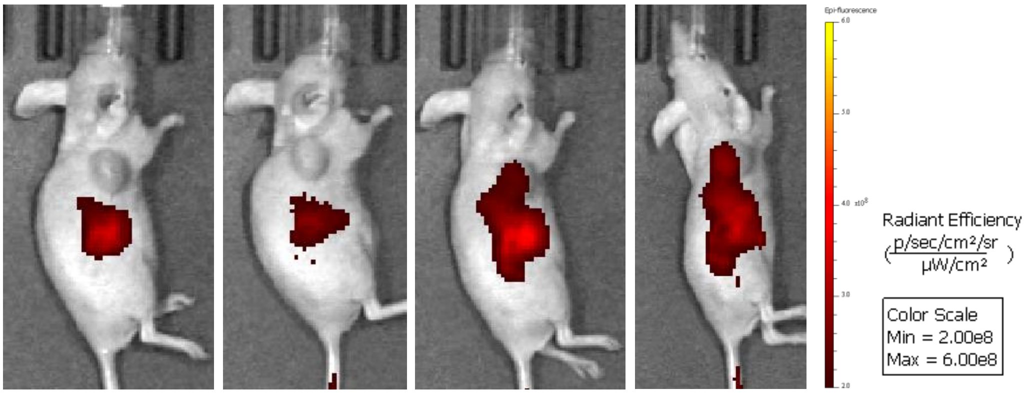

Fig. S14 Additional time point images of **3** from Fig. 9.

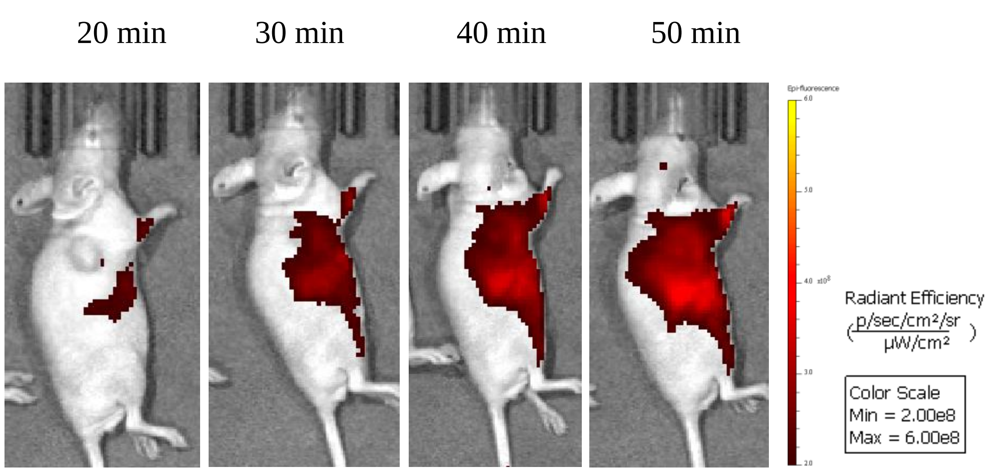

Fig. S15 Excised tumour fluorescence quantification for RGD competitive binding study.

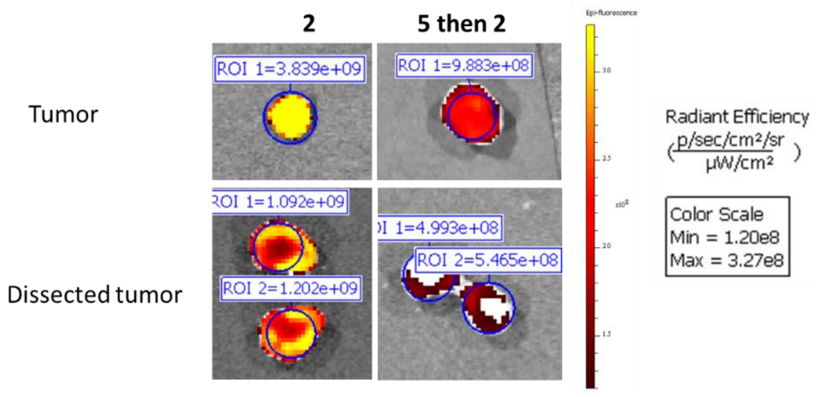

Synthetic Procedures

Synthesis of fluorophore 1.

Fluorophore **1** was synthesized according to the previously reported procedure.[1]

Synthesis of fluorophore 4.

Fluorophore **4** was synthesized according to the previously reported procedure.[1]

Synthesis of fluorophore 6.

Fluorophore **4** was synthesized according to the previously reported procedure.[2]

Synthesis of fluorophore 6.

Fluorophore **4** was synthesized according to the previously reported procedure.[2]

Synthesis of fluorochrome 7.

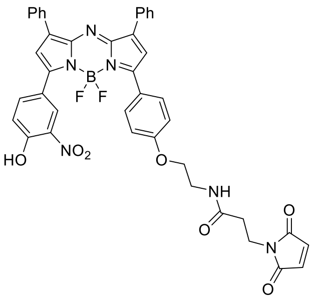

A solution of **6** (50 mg, 0.07 mmol) in acetonitrile (1.2 mL) was heated under reflux for 5 min. A solution of KNO_3 (7.8 mg, 0.08 mmol) and $KHSO_4$ (19 mg, 0.14 mmol) in water (0.2 mL) was added and the mixture was heated under reflux for 5 min. The suspension was cooled to room temperature (rt) and partitioned between AcOEt (60 mL) and water (40 mL). The organic phase was washed with water (2 × 40 mL), brine (2 × 50 mL), dried over Na_2SO_4, filtered and evaporated to dryness. The crude was purified by silica gel chromatography, eluting with CH_2Cl_2: MeOH (50: 1) to yield the product **7** as a dark solid (35 mg, 67%). mp: 164-165 °C; ^{1}H NMR (400 MHz, DMSO-d_6): 11.94 (brs, 1H), 8.69 (d, J = 2.1 Hz, 1H), 8.32 - 8.22 (m, 4H), 8.21 - 8.13 (m, 4H), 7.75 (s, 1H), 7.60 (s, 1H), 7.59-7.46 (m, 6H), 7.29 (d, J = 8.9 Hz, 1H), 7.16 (d, J = 8.9 Hz, 2H), 6.96 (s, 2H), 4.12 (t, J = 5.4 Hz, 2H), 3.63 (t, J = 7.2 Hz, 2H), 3.44 (q, J = 5.5 Hz, 2H), 2.38 (t, J = 7.2 Hz, 2H) ppm. ^{13}C NMR (100 MHz, DMSO-d_6): 170.8, 169.9, 161.9, 159.8, 154.1, 153.6, 145.6, 143.8, 143.6, 141.4, 137.3, 135.6, 134.6, 132.4, 132.0, 131.5, 130.0, 129.6, 129.3, 129.1, 128.82, 128.79, 126.7, 122.8, 122.0, 121.0,

119.5, 119.0, 115.1, 66.8, 38.1, 34.1, 34.1 ppm. HRMS calcd. for $C_{41}H_{30}BN_6O_7F_2$ $[M-H]^-$:767.2249; found 767.2263.

Synthesis of RGD NIR-AZA conjugate 2

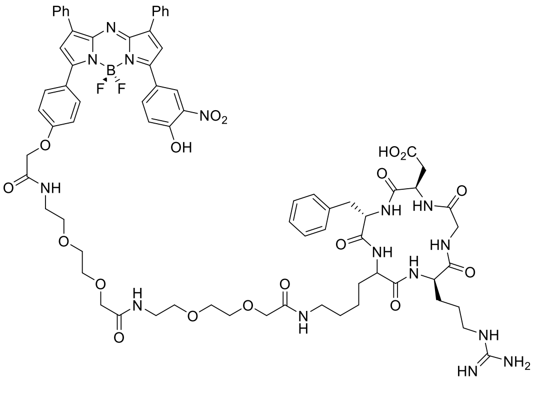

A vial containing cyclic peptide cyclo[Arg-Gly-Asp-D-Phe-Lys(PEG-PEG-NH_2)] **5** (5 mg, 0.0056 mmol) (purchased from Peptides International, cat. no. PCI-3696-PI) was flushed with N_2 and its contents were dissolved with 600 µL anhydrous DMSO. The vial was sonicated and **5** in DMSO was then added via syringe to a separate oven dried 1.5 mL screw cap (with a septum) N_2 flushed vial containing activated ester **4** (4.5 mg, 0.0062 mmol) and magnetic stir bar. The vial originally containing **5** was rinsed with an additional 100 µL of anhydrous DMSO with this was also added to the reaction vial and the reaction set to stir. Additionally, DIPEA (1.2 µL, 0.0067 mmol) was added and the reaction was followed by HPLC (ACN: H_2O 60:40 with 10 mM NH_4HCO_3 - flow 0.6 mL/min). After 4 hours the reaction mixture was judged to have gone to completion and the reaction was diluted with H_2O (10 mL) and lyophilized. The crude was dissolved in 40:60; ACN: H_2O with 10 mM NH_4HCO_3, filtered through a PTFE 0.45 µM syringe filter and the resulting green solution was purified by reverse phase semi prep chromatography (YMC-Triart Phenyl, 10 × 150 mm I.D. S-5 µm, 12 nm; - eluant ACN: H_2O 40:60 - flow 3 mL/min). Pure fractions (analyzed by HPLC) of **2** were combined and reduced to dryness by freeze drying to afford each construct as a light green powder. The residue was dissolved in sodium bicarbonate buffer (10 mM) and transferred to an Eppendorf where it was carefully acidified with 1M HCl causing **2** to precipitate. **2** was centrifuged to remove any salts and resuspended in HPLC grade H_2O before being centrifuged again with this process been carried out a total of 3 times. The final pellet was freeze-dried to afford **2** as the HCl salt (7.3 mg, 85 %). ^1H NMR (500 MHz, DMSO-*d6*) δ: 12.24 (brs, 1H), 8.94 (s, 1H), 8.38 (dd, *J* = 7.3, 4.3 Hz, 1H), 8.25-8.21 (m, 3H), 8.19 (t, *J* = 5.7 Hz, 1H), 8.13-8.07 (m, 3H), 8.05-7.98 (m, 4H), 7.96 (s, 1H), 7.69-7.64 (m, 3H), 7.54 (t, *J* = 7.4 Hz, 2H), 7.50-7.46 (m, 3H), 7.42 (t, *J* = 5.6 Hz, 1H), 7.37 (t, *J* = 7.3 Hz, 1H), 7.27-7.20 (m, 3H), 7.20-7.12 (m, 3H), 7.08 (d, *J* = 8.9 Hz, 2H), 6.65 (br s, 1H), 4.66-4.62 (m, 1H), 4.61 (s, 2H), 4.48-4.42 (m, 1H), 4.18-4.12 (m, 1H), 4.05 (dd, *J* = 15.0, 7.7 Hz, 1H), 3.95-3.89

(m, 1H), 3.89 (s, 2H), 3.85 (s, 2H), 3.58 (s, 4H), 3.55 (s, 4H), 3.50 (t, *J* = 5.9 Hz, 2H), 3.45 (t, *J* = 6.0 Hz, 2H), 3.37-3.34 (m, 2H), 3.30-3.27 (m, 2H), 3.24 (dd, *J* = 15.0, 4.1 Hz, 1H), 3.12-3.06 (m, 2H), 3.05-2.99 (m, 2H), 2.92 (dd, *J* = 13.5, 8.2 Hz, 1H), 2.80 (dd, J = 13.5, 6.0 Hz, 1H), 2.70 (dd, *J* = 16.2, 8.5 Hz, 1H), 2.37 (dd, *J* = 16.2, 5.9 Hz, 1H), 1.76-1.67 (s, 1H), 1.59-1.27 (m, 7H), 1.06-0.98 (m, 2H) ppm. HRMS calcd. for $C_{73}H_{85}BN_{15}O_{18}F_2$ $[M+H]^+$: 1508.6258; found 1508.6302 Da.

Synthesis of iRGD peptide 8

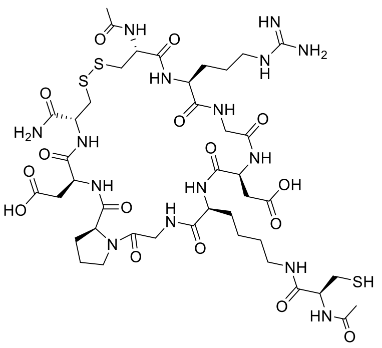

Automated Fmoc-SPPS was carried out on a Biotage Initiator+Alstra microwave peptide synthesizer equipped with an inert gas manifold. General synthetic protocols for Fmoc deprotection and capping were carried out in accordance with the manufacturer′s specifications. Standardized amino acid couplings were performed for 20 min at 50 °C under microwave irradiation in the presence of amino acid (0.3 m in DMF), Oxyma Pure (ethyl (hydroxyimino)cyanoacetate, 0.5 m in DMF), and N,N′-diisopropylcarbodiimide (0.5 m in DMF). The resin bound peptide Fmoc-Cys(Acm)-Arg(Pbf)-Gly-Asp(OtBu)-Lys(Alloc)-Gly-Pro-Asp(OtBu)-Cys(Acm)-NH−Resin was assembled on Rink amide resin (approx. 200 µM loading) using the automated peptide assembly protocol described above. After assembly, peptide cyclisation was achieved by treatment of the resin with a mixture of thallium trifluoroacetate (2 equiv.) in DMF (5 mL) for 2 hrs before washing with MeOH (3 × 5 mL), DMF (3 × 5 mL) and CH_2Cl_2 (3 × 5 mL). The cyclised on-resin peptide Fmoc-cyclo[Cys(~)-Arg(Pbf)-Gly-Asp(OtBu)-Lys(Alloc)-Gly-Pro-Asp(OtBu)-Cys(~)]-NH−Resin was treated with 20 % piperidine/DMF (2 × 3 mL, 3 min) to remove the Fmoc protecting group and washed with DMF (× 5), CH_2Cl_2 (× 5) and DMF (× 5) before N-terminal acetylation with 20 % acetic anhydride/pyridine (3 × 4 min) followed by washing with DMF (× 5), CH_2Cl_2 (× 5) and DMF (× 5) afforded acetyl-cyclo[Cys(~)-Arg(Pbf)-Gly-Asp(OtBu)-Lys(Alloc)-Gly-Pro-Asp(OtBu)-Cys(~)]-NH−Resin. Alloc-deprotection of the Lys residue was performed by swelling of the resin at RT for 15 min in $CHCl_3$/morpholine/acetic acid (90:5:5). Tetrakis(triphenylphosphine)palladium (1.05 equiv relative

to peptide) was added to the suspension, and the syringe was shielded from light and agitated for 2 hrs. The resin was drained then washed with $CHCl_3$ (× 5) and a palladium-chelating cocktail (DMF/diethyldithiocarbamic acid-3-water/triethylamine 25 mL:225 mg:250 μL). Traces of the chelating cocktail were removed by a basic wash (0.5 % triethylamine in DMF, 5 × 5 mL). The resin was then washed with MeOH (× 5), DMF (× 5), CH_2Cl_2 (× 5), and DMF (× 5). A solution of Fmoc-Cys(Trt) (4 equiv), PyBOP (4 equiv.) and NMM (8 equiv.) in DMF (final concentration 0.1 m) was added to the resin. After 1 h, the resin was washed with DMF (× 5), CH_2Cl_2 (× 5) and DMF (× 5). The resin was again treated with 20 % piperidine/DMF (2 × 3 mL, 3 min) and washed with DMF (× 5), CH_2Cl_2 (× 5) and DMF (× 5) before subsequent acetylation with 20 % acetic anhydride/pyridine (3 × 4 min) followed by washing with DMF (× 5), CH_2Cl_2 (× 5), DMF (× 5) and CH_2Cl_2 (× 5) afforded Acetyl-cyclo[Cys(~)-Arg(Pbf)-Gly-Asp(OtBu)-Lys(Acetyl-Cys(Trt))-Gly-Pro-Asp(OtBu)-Cys(~)]-NH−Resin. Peptide cleavage was carried out by adding a mixture of TFA, triisopropylsilane (TIS), and water (90:5:5 v/v/v) to the resin. After 2 hrs, the resin was washed with TFA (3 × 2 mL). The combined cleavage and TFA wash solutions were concentrated under a stream of nitrogen to <5 mL before diethyl ether (30 mL) was added to precipitate the peptide which was isolated by centrifugation at 4000 rpm for 5 min. The residue was lyophilised and analysed by LC-HRMS. HRMS (ESI): m/z calcd for $C_{42}H_{68}N_{15}O_{16}S_3$: 1134.4125; $[M+H]^+$; found: 1134.4135.

Synthesis of iRGD NIR-AZA conjugate 3.

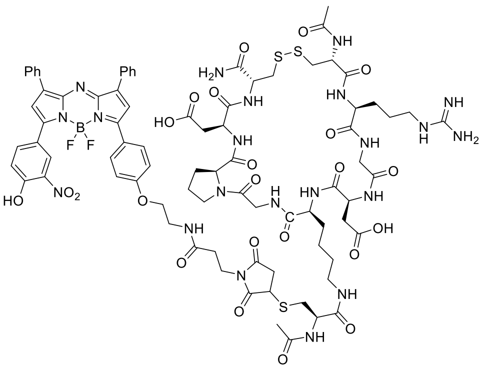

A vial containing cyclic iRGD peptide **8** (8 mg, 0.007 mmol) was flushed with N_2 and its contents were dissolved with 1 mL anhydrous DMSO. The vial was sonicated and the solution of **8** was then added via syringe to an oven dried 1.5 mL screw cap (with a septum) N_2 flushed vial containing **7** (4.5 mg, 0.0059 mmol) and a magnetic stir bar. The vial originally containing **8** was rinsed with an additional 100 μL of anhydrous DMSO, this added to the reaction vial and the reaction stirred at rt. The reaction was followed by HPLC (ACN: H_2O 40:60 with 10 mM NH_4HCO_3 - flow 1 mL/min). After 30 min the reaction mixture was judged to have gone to completion and the reaction was diluted

with H_2O (10 mL) and lyophilized. The crude was dissolved in 40:60; ACN:H_2O with 10 mM NH_4HCO_3, filtered through a PTFE 0.45 μM syringe filter and the resulting green solution was purified by reverse phase semi prep chromatography (YMC-Triart Phenyl, 10 × 150 mm I.D. S-5 μm, 12 nm; - eluant ACN: H_2O 40:60 - flow 3 mL/min). Pure fractions (analyzed by HPLC) of **3** were combined and reduced to dryness by freeze drying. The residue was further purified by Sephedex G-25 desalting column to remove salts. The final pure green fraction was freeze-dried to afford **3** as a light green powder. (7.9 mg, 71%). ^{1}H NMR (400 MHz, DMSO-*d6*): 9.02 (s, 1H), 8.32-8.19 (m, 5H), 8.10 (d, *J* = 7.5 Hz, 4H), 7.97 (d, *J* = 8.6 Hz, 2H), 7.54 (q, *J* = 8.3, 7.8 Hz, 3H), 7.50-7.44 (m, 2H), 7.33 (t, *J* = 7.4 Hz, 1H), 7.27 (brs, 1H), 7.22 (brs, 1H), 7.11-7.05 (m, 3H), 6.46 (d, *J* = 9.7 Hz, 2H) 4.50-0.76 (peptide peaks with alkyl CH_2 from fluorophore overlapped) ppm. MS (ESI): m/2 calcd. for $C_{83}H_{96}BN_{21}O_{23}F_2S_3$ $[M-2H]^{2-}$: 950.3; found 949.1 Da. HRMS (ESI): m/z calcd for $C_{83}H_{99}BN_{21}O_{23}F_2S_3$ $[M+H]^+$: 1902.6446; found: 1902.6421 Da.

Synthesis of iRGD NIR-AZA conjugate 10

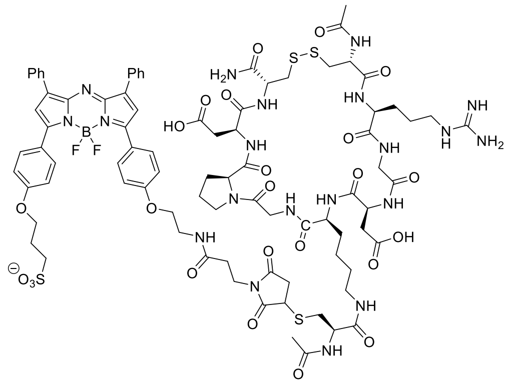

A vial containing cyclic iRGD peptide **8** (8 mg, 0.007 mmol) was dissolved with 1 mL degassed PBS (pH=7.2, with 10 uL DMSO). The vial was sonicated for five minutes and the solution of **8** then added via syringe to an oven dried 1.5 mL screw cap (with a septum) N_2 flushed vial containing **9** (5 mg, 0.0059 mmol) and magnetic stir bar. The vial originally containing **8** was rinsed with an additional 100 μL of PBS sonicated for five minutes, added to the reaction vial and the reaction stirred at rt. The reaction was followed by HPLC (ACN:H_2O 40:60 with 10 mM NH_4HCO_3 - flow 1 mL/min). After 5 min the reaction mixture was judged to have gone to completion and the reaction was purified by reverse phase semi prep chromatography (YMC-Triart Phenyl, 10 × 150 mmI.D. S-5 μm, 12 nm; - eluant ACN: H_2O 40:60 - flow 3 mL/min). Pure fractions (analyzed by HPLC) of **10** were combined and reduced to dryness by freeze drying. The residue was further purified by Sephedex G-25 desalting column to remove salts. The final pure green fraction was freeze-dried to afford **10** as a light green

powder. (8.7 mg, 75%). ^{1}H NMR (400 MHz, DMSO-*d6*): 8.32 (brs, 2H), 8.26-8.05 (m, 8H), 7.64 (d, *J* = 14.3 Hz, 2H), 7.55 (t, *J* = 7.4 Hz, 2H), 7.50-7.42 (m, 3H), 7.28-7.20 (m, 2H), 7.18-7.08 (m, 3H), 7.01-6.94 (m, 1H), 4.23 (t, J = 6.5 Hz, 2H), 4.05 (t, *J* = 6.8 Hz, 2H), 4.75-0.81(peptide peaks with alkyl CH_2 from fluorophore overlapped) ppm. HRMS m/2 calcd. for $C_{86}H_{103}BN_{20}O_{24}F_2S_4$ $[M-2H]^{2-}$:988.3204; found 988.8189 Da.

References

1. M. Grossi, M. Morgunova, S. Cheung, D. Scholz, E. Conroy, M. Terrile, A. Panarella, J. C. Simpson, W. M. Gallagher and D. F. O'Shea, *Nat. Commun*. 2016, **7**, 10855-10867.

2. D. Wu, S. Cheung, M. Devocelle, L. Zhang, Z. L. Chen and D. F. O'Shea, *Chem. Commun*., 2015, **51**, 16667-16670

Analytical data for PEG NIR-AZA conjugate 1[1]

^{1}H NMR (400 MHz, DMSO-d_6)

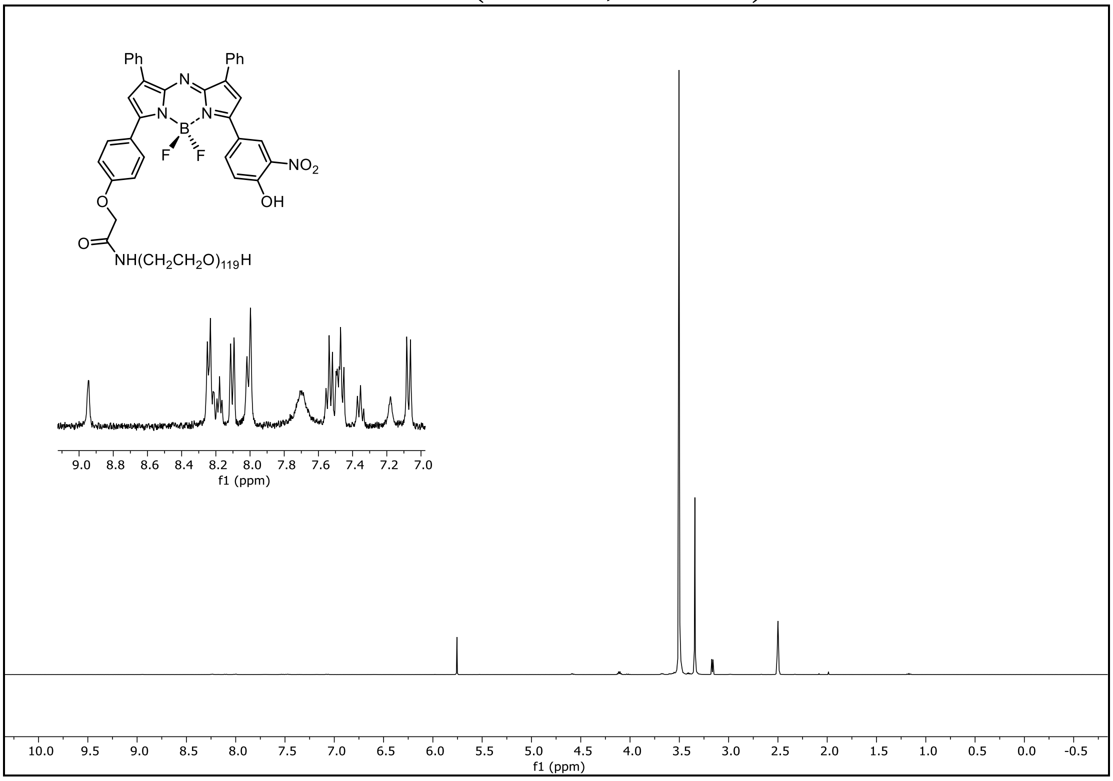

MALDI MS of **1**

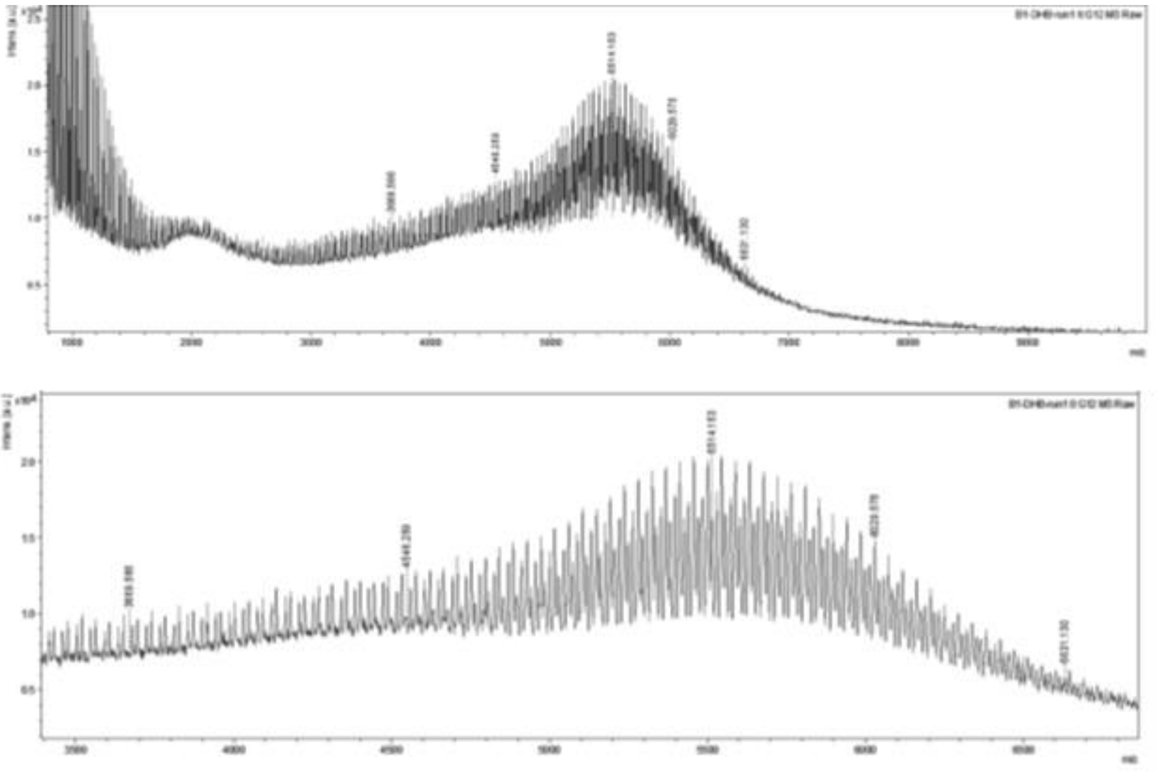

Analytical Data for Fluorochrome 7.

^{1}H NMR (400 MHz, DMSO-d_6)

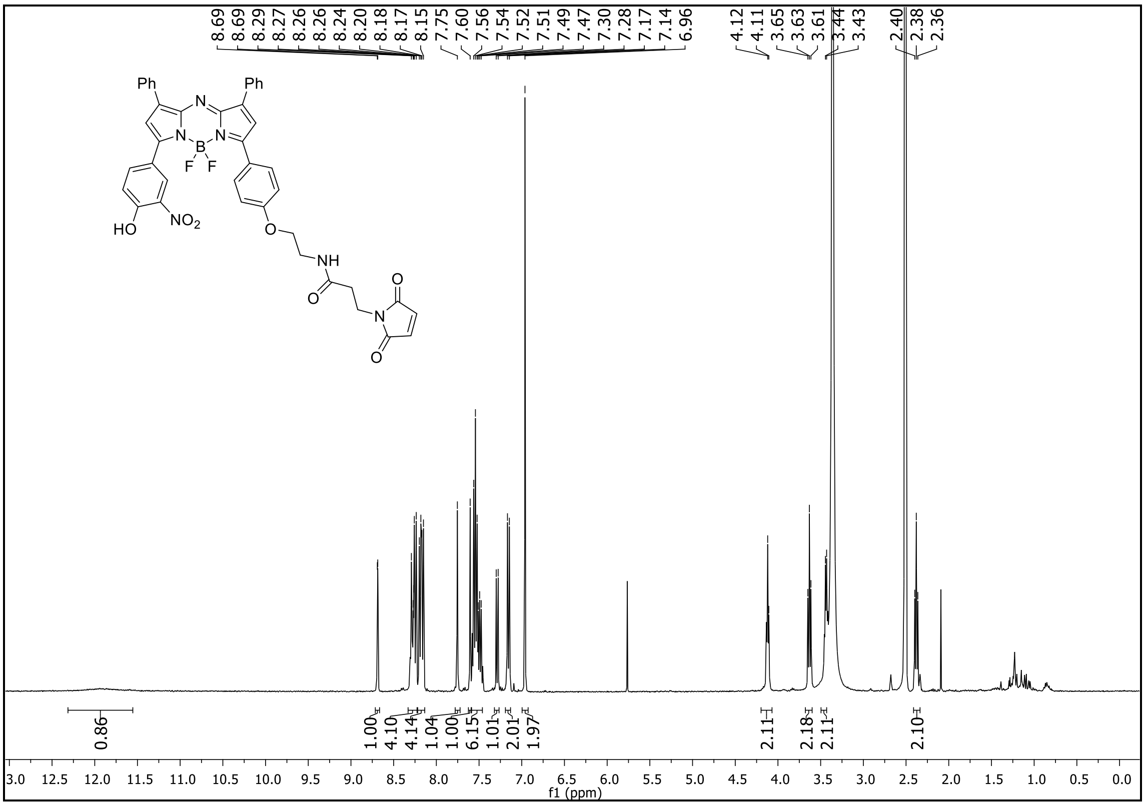

^{13}C NMR (100 MHz, DMSO-d_6)

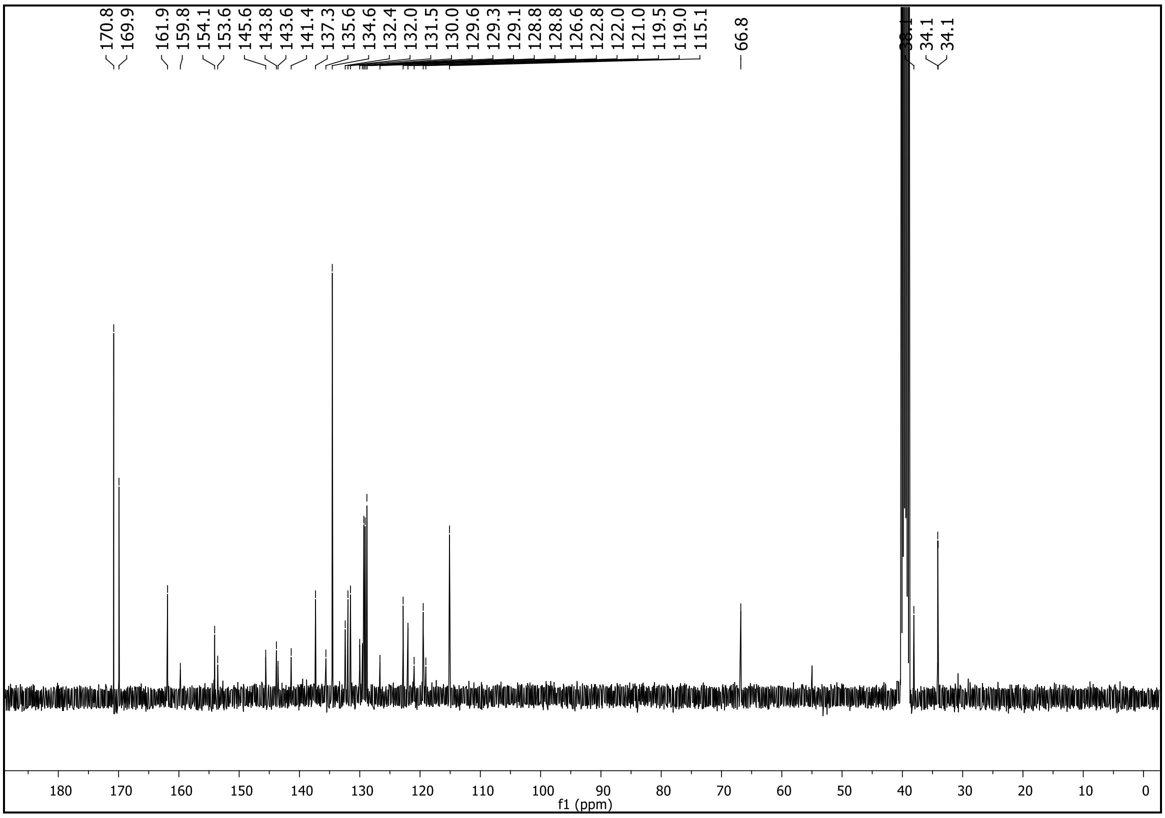

^{19}F NMR (375 MHz, DMSO-d_6)

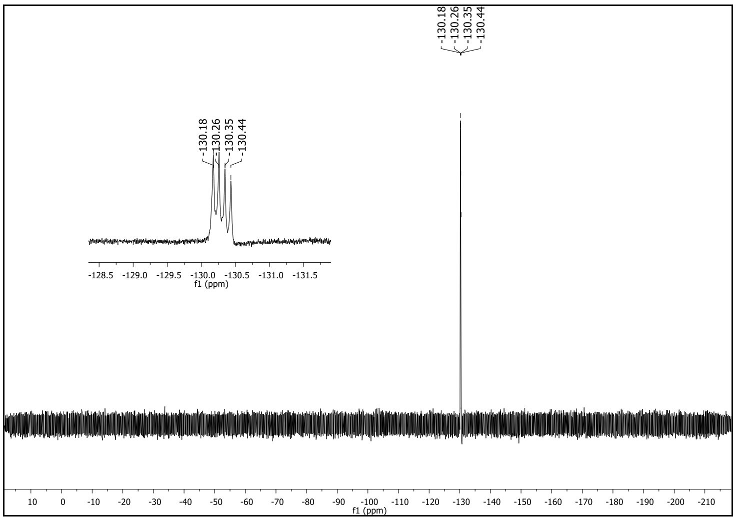

^{1}H COSY (DMSO-d_6)

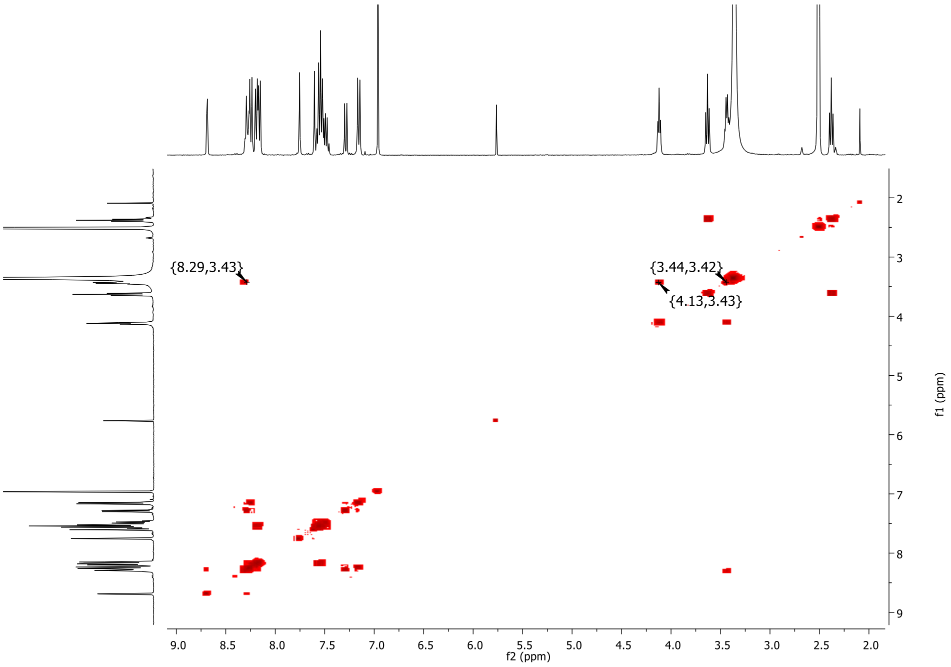

HRMS (ESI)

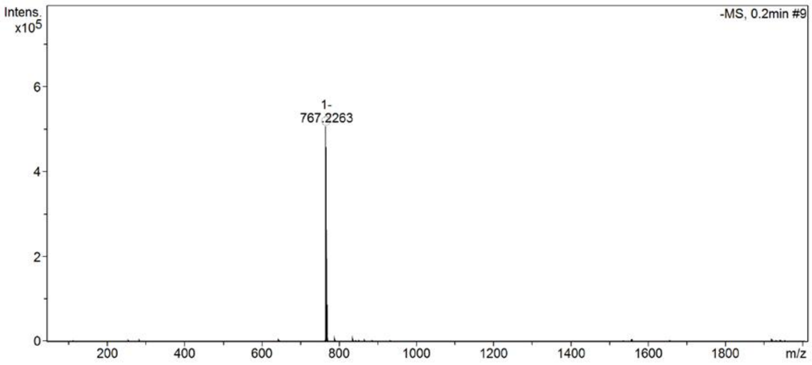

Meas. m/z	#	Ion Formula	m/z	err [mDa]	err [ppm]	rdb	N-Rule	e⁻ Conf	mSigma
767.226313	1	C41H30BF2N6O7	767.224962	1.4	1.8	29.5	ok	even	5.0

Analytical Data for cRGD NIR-AZA conjugate 2

^{1}H NMR (500 MHz, DMSO-d_6)

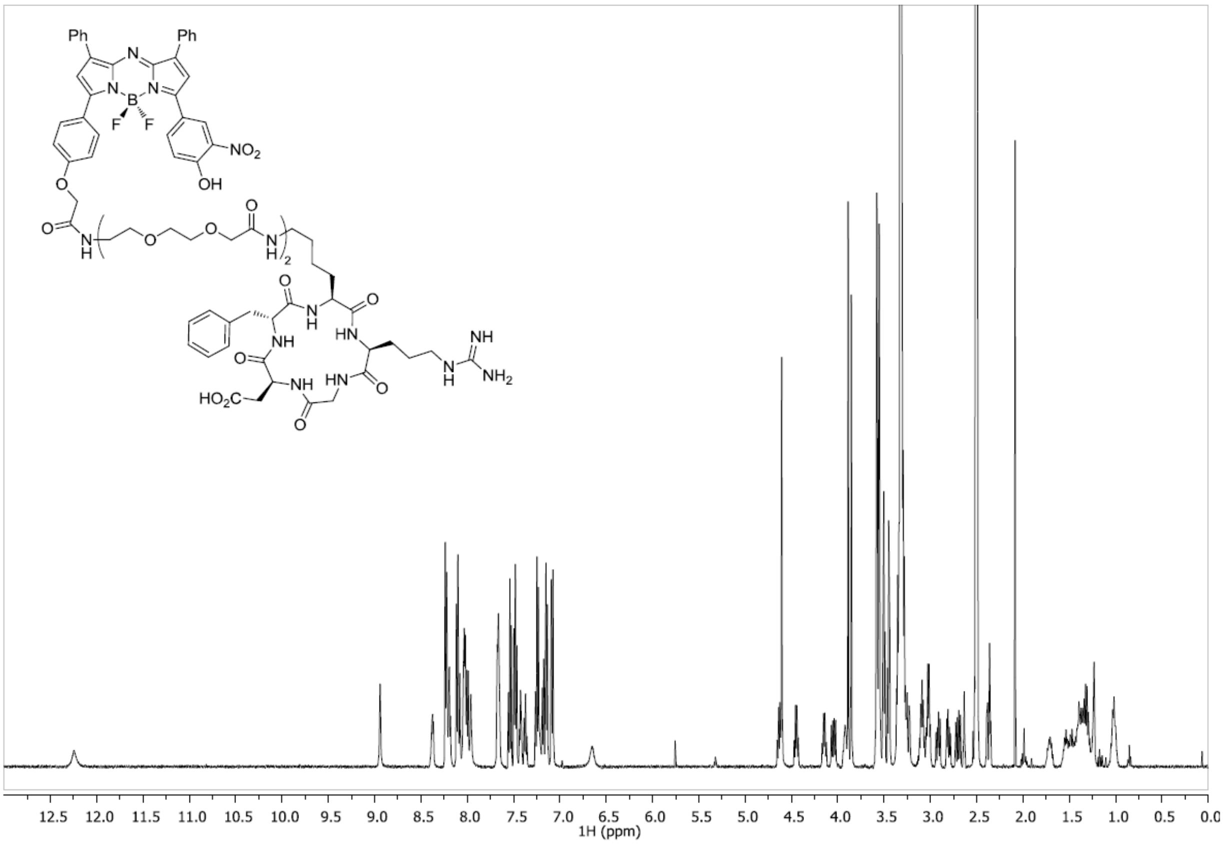

^{1}H COSY (DMSO-d_6)

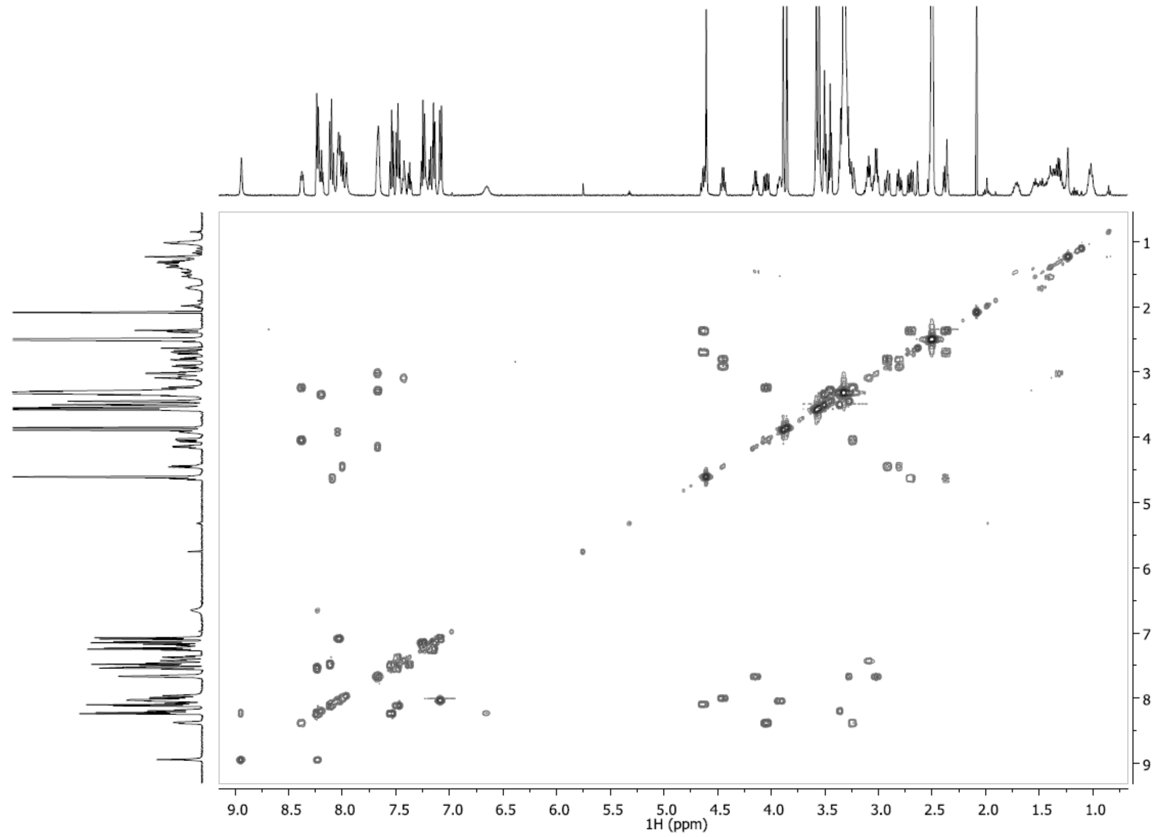

Mass spectrum

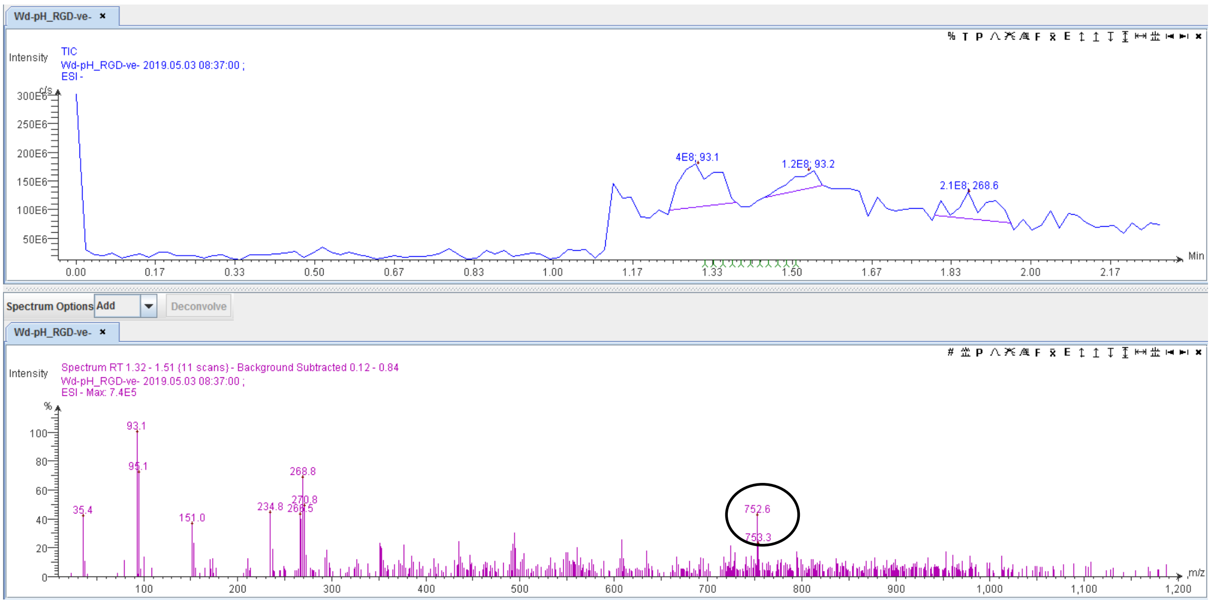

Mass spectrum of RGD conjugate **2** showing peaks corresponding to $[M-2H]^{2-}$ ion.

HPLC trace of purified 2

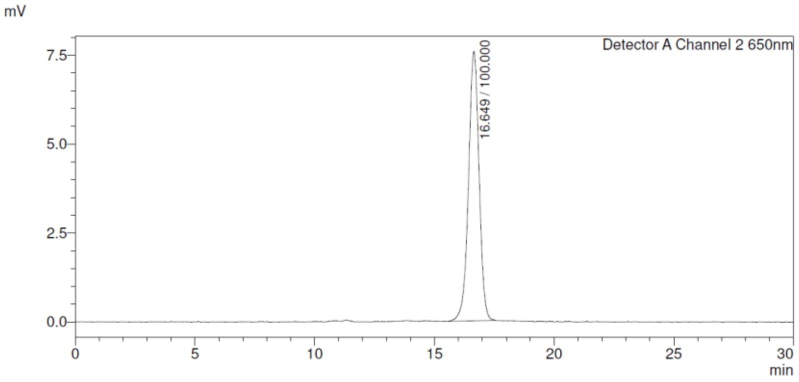

Condition: RP-HPLC with YMC triart phenyl column and size: 150 × 4.6 mm I.D., particle size: S-5μm, 12 nm hole, detection method: UV-Vis and wavelength for detection: 600 nm and 650 nm. Eluent CH_3CN:H_2O = 40:60 (10 mM NH_4HCO_3) with a flow rate at 1 mL/min.

Analytical Data for iRGD peptide 8

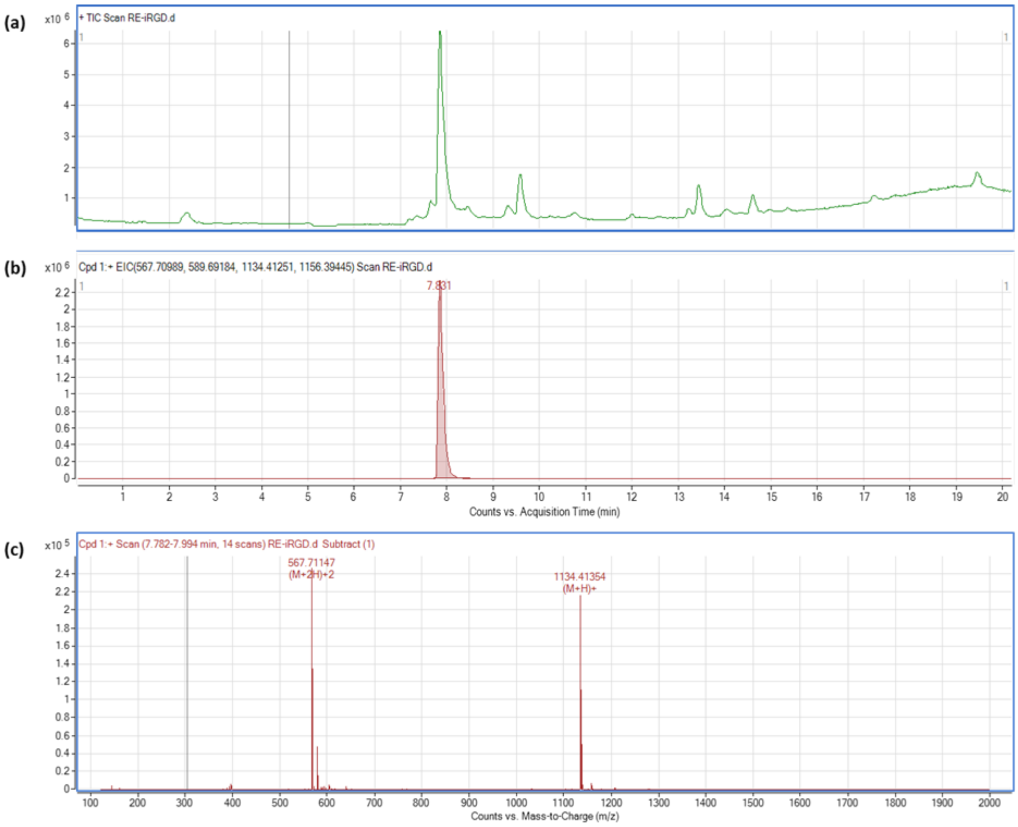

(a) HPLC Total Ion Chromatogram (TIC) for iRGD (b) Extracted Ion Chromatogram (EIC) of iRGD at 7.83 minutes. (c) High resolution mass spectrum (HRMS) of iRGD **8** showing peaks corresponding to $[M+H]^{+}$ and $[M+2H]^{2+}$ ions.

Analytical Data for iRGD NIR-AZA Conjugate 3

^{1}H NMR (400 MHz, DMSO-d_6)

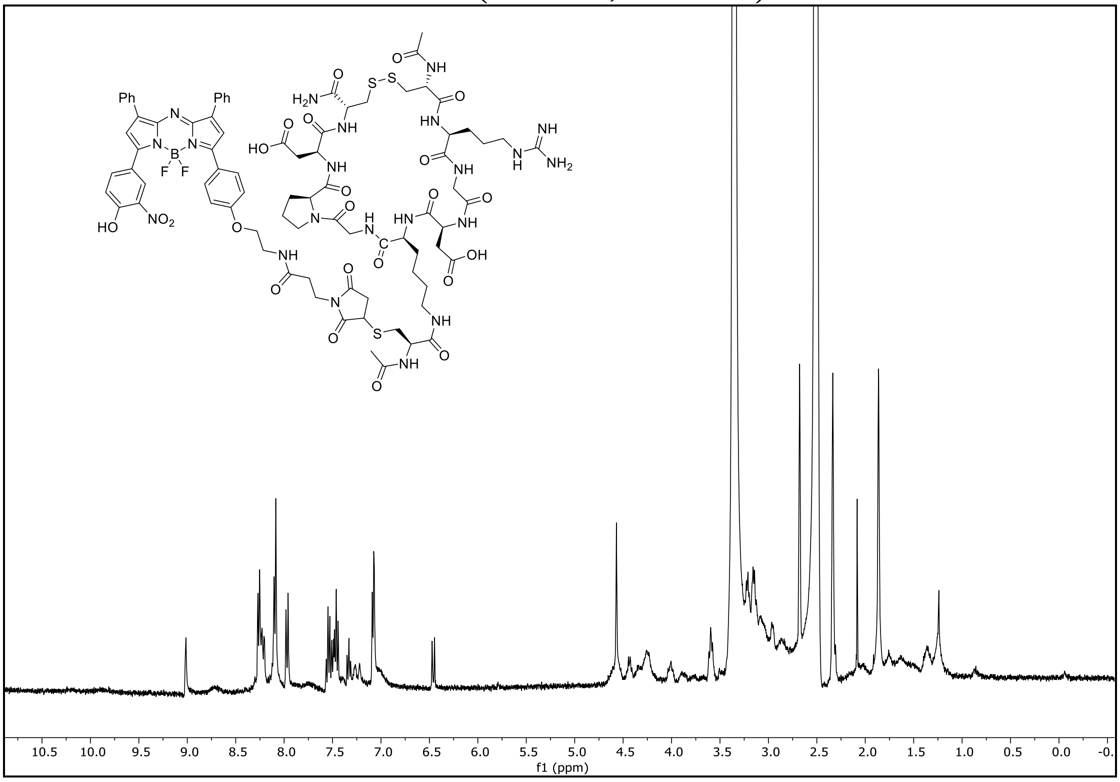

^{1}H NMR spectra of **7** to **3** with significant maleimide peak change (shown in arrow)

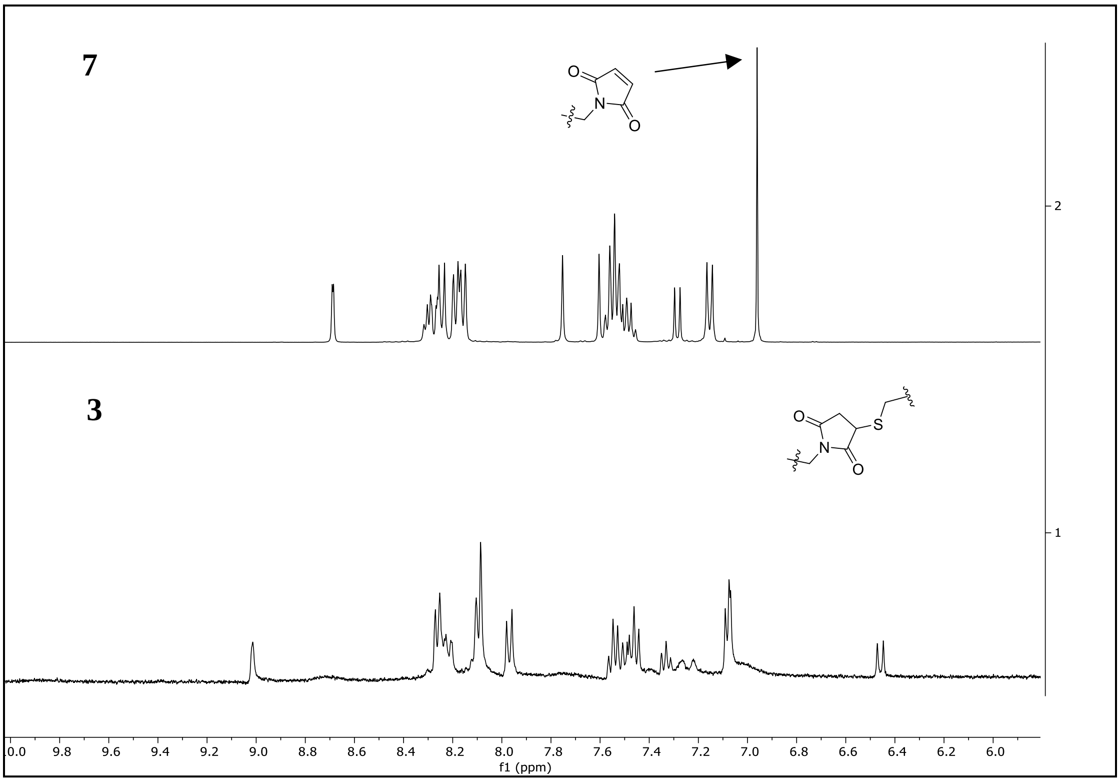

Mass spectrum

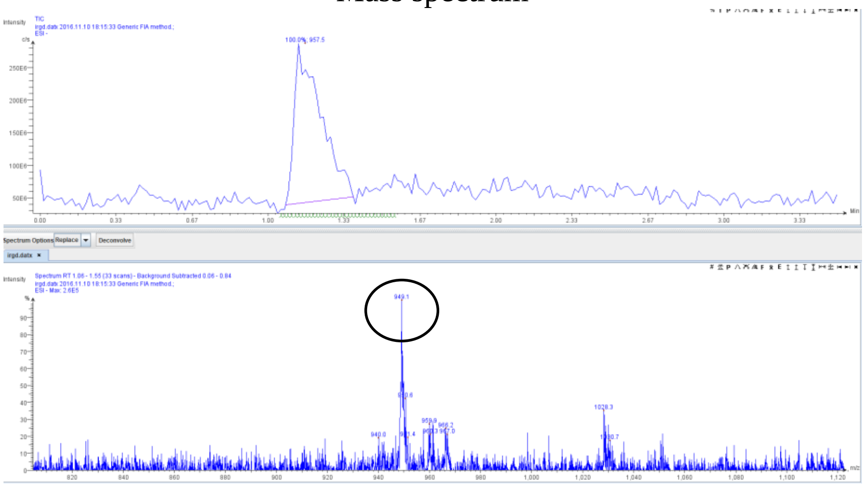

Mass spectrum of iRGD conjugate **3** showing peaks corresponding to $[M-2H]^{2-}$ ion.

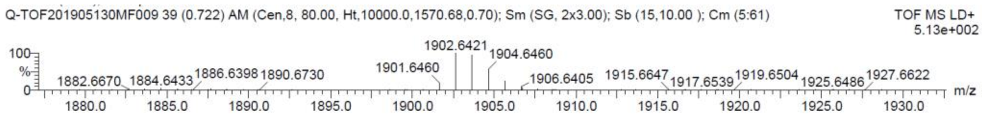

Minimum:				-1.5			
Maximum:		5.0	20.0	400.0			
Mass	Calc. Mass	mDa	PPM	DBE	i-FIT	i-FIT (Norm)	Formula
1902.6421	1902.6446	-2.5	-1.3	44.5	55.7	0.0	C83 H99 B N21 O23 S3 F2

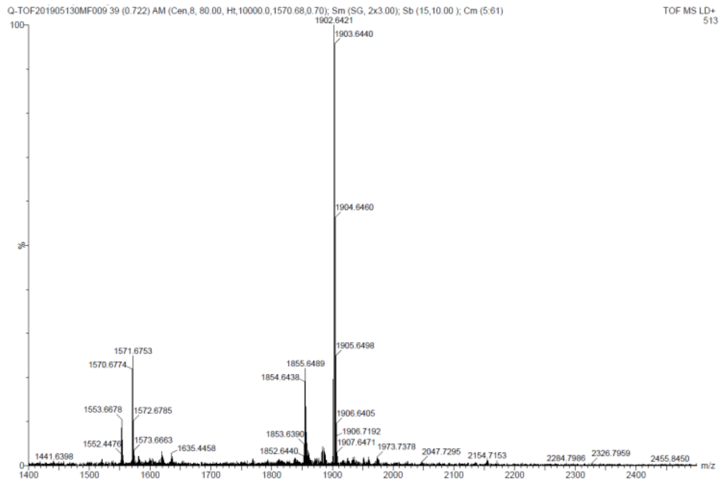

HPLC trace of purified **3**

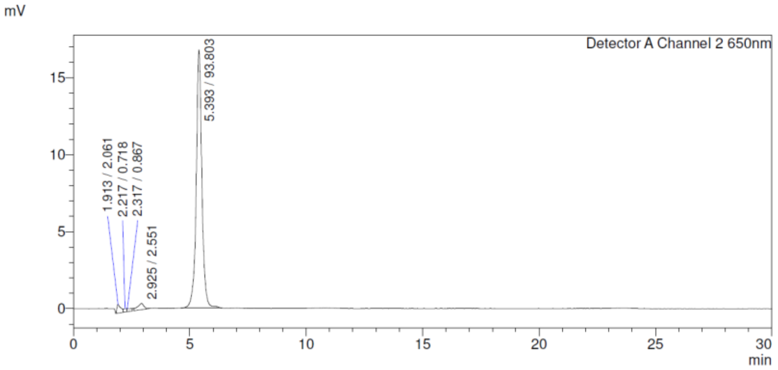

Condition: RP-HPLC with YMC triart phenyl column and size: 150 × 4.6 mm I.D., particle size: S-5µm, 12 nm hole, detection method: UV-Vis and wavelength for detection: 600 nm and 650 nm. Eluent CH_3CN :H_2O =40:60 (10 mM NH_4HCO_3) with a flow rate at 1 mL/min.

Analytical Data for iRGD NIR-AZA Conjugate 10

^{1}H NMR (400 MHz, DMSO-d_6)

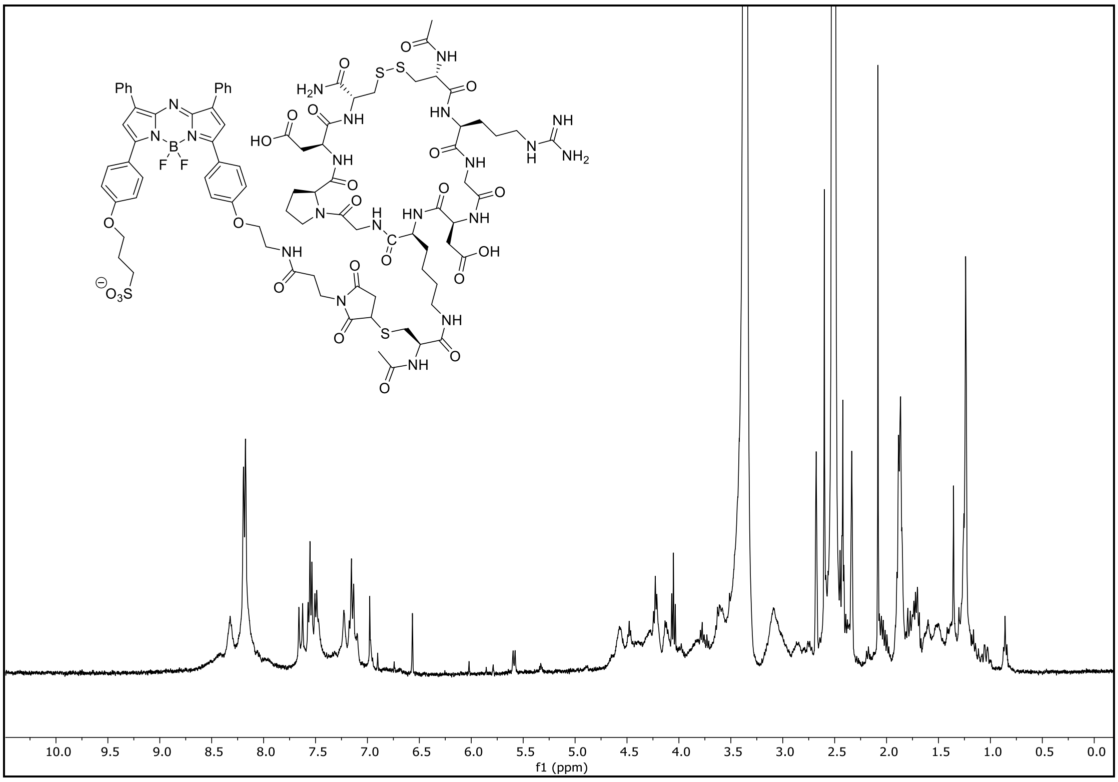

Mass spectrum

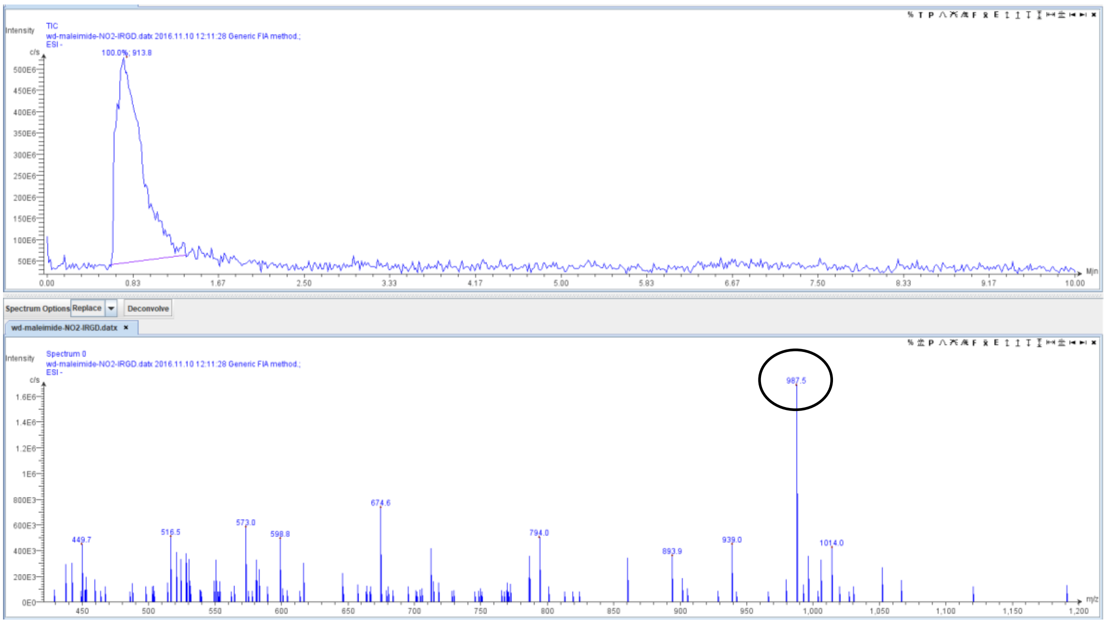

Mass spectrum of iRGD conjugate **10** showing peaks corresponding to $[M-2H]^{2-}$ ion.

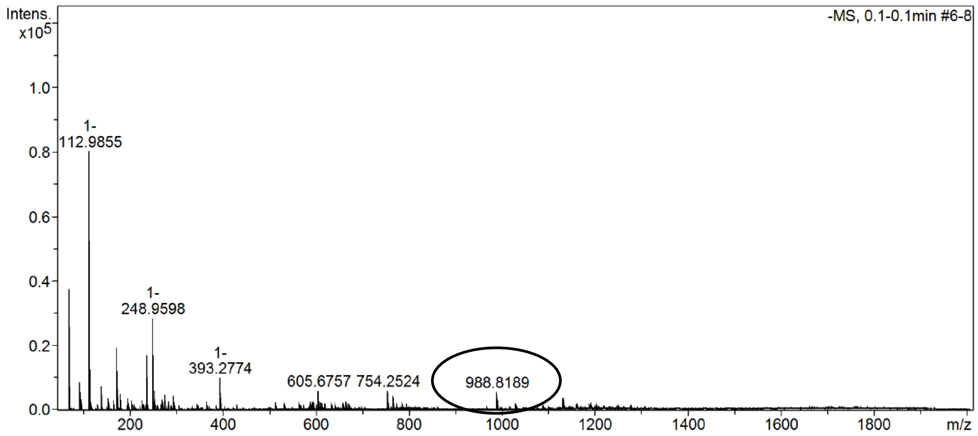

HR mass spectrum of iRGD conjugate **10** showing $[M-2H]^{2-}$ ion.

HPLC trace of purified **10**

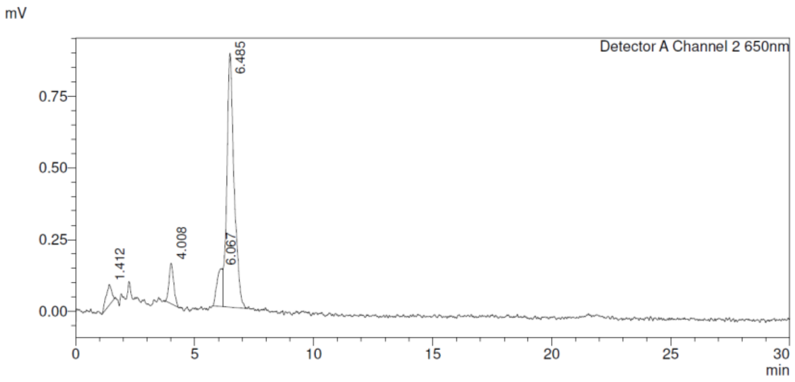

Condition: RP-HPLC with YMC triart phenyl column and size: 150 × 4.6 mm I.D., particle size: S-5µm, 12 nm hole, detection method: UV-Vis and wavelength for detection: 600 nm and 650 nm. Eluent CH_3CN:H_2O =40:60 (10 mM NH_4HCO_3) with a flow rate at 1 mL/min.
